# Dynamic Instrumental and Sensory Methods Used to Link Aroma Release and Aroma Perception: A Review

**DOI:** 10.3390/molecules28176308

**Published:** 2023-08-29

**Authors:** Jean-Luc Le Quéré, Rachel Schoumacker

**Affiliations:** Centre des Sciences du Goût et de l’Alimentation (CSGA), CNRS, INRAE, Institut Agro, Université de Bourgogne, F-21000 Dijon, France

**Keywords:** in vivo, aroma release, aroma perception, dynamic methods, nosespace, DIMS, TI, TDS, TCATA, APCI-MS, PTR-MS

## Abstract

Perception of flavor is a dynamic process during which the concentration of aroma molecules at the olfactory epithelium varies with time as they are released progressively from the food in the mouth during consumption. The release kinetics depends on the food matrix itself but also on food oral processing, such as mastication behavior and food bolus formation with saliva, for which huge inter-individual variations exist due to physiological differences. Sensory methods such as time intensity (TI) or the more-recent methods temporal dominance of sensations (TDS) and temporal check-all-that-apply (TCATA) are used to account for the dynamic and time-related aspects of flavor perception. Direct injection mass spectrometry (DIMS) techniques that measure in real time aroma compounds directly in the nose (nosespace), aimed at obtaining data that reflect the pattern of aroma release in real time during food consumption and supposed to be representative of perception, have been developed over the last 25 years. Examples obtained with MS operated in chemical ionization mode at atmospheric or sub-atmospheric pressure (atmospheric pressure chemical ionization APCI or proton-transfer reaction PTR) are given, with emphases on studies conducted with simultaneous dynamic sensory evaluation. Inter-individual variations in terms of aroma release and their relevance for understanding flavor perception are discussed as well as the evidenced cross-modal interactions.

## 1. Introduction

Among the criteria that determine food choice by the consumer, sensory properties of foods are of prime importance. Among these, flavor is an essential element in perceived food quality. Flavor is sensed by the integration of sensations in the brain, including possible cognitive interactions. Flavor perceptions occur in the mouth when foods are eaten and can be defined as taste and aroma; feeling of pain, heat, and cold (chemesthesis or trigeminal sensitivity); and tactile sensation. However, sensory food qualities are generally experienced as a unique perception commonly called “taste”. This familiar “taste” is a holistic perception of at least aroma and taste, generally called “flavor perception” [1]. Aroma plays a major role in the overall flavor, as easily demonstrated by the difficulties encountered when trying to identify a particular flavor if the airflow through the nose is prevented. The molecular sensory science approach, so-called “sensomics” [2], or equivalent procedures have constituted a major breakthrough in identifying aroma-active compounds in food. Combining the extraction of volatile organic compounds (VOCs), identification of odor-active compounds among them, and a validation step through recombination and/or omission protocols, this approach constitutes the state of the art for identifying aroma-active components in food [2].

However, relating aroma compounds’ composition to aroma perception by humans is not straightforward. Poor correlations are often found between all the aroma compounds identified in a food and the sensory perception by a consumer eating this food. Monitoring specific odor-active compounds with their known individual aroma quality does not necessarily indicate their actual contribution to the overall flavor. In fact, it is still not completely understood how the various components, their interactions, and their interactions with physiology combine to produce a sensory impression [3,4]. Moreover, a significant correlation between analytical and sensory data does not necessary imply a causal relationship between the two [5]. Food aroma compounds elicit a transduction cascade after interacting primarily in a combinatorial code with ca. 400 olfactory receptors in humans. A few hundreds of foodborne volatiles lead to a multitude of odor and aroma perceptual qualities [6]. Moreover, in a sensory evaluation of foodstuffs, flavor is evaluated in a complex mixture context where interactions with the food matrix occur [7] and where masking or enhancing effects may influence the overall sensory perception [8,9].

Perception of flavor is a dynamic process [10] changing in both the short and long term, and time dimension is de facto implicit in perceptual experience [8]. During food consumption, aroma compounds are released progressively from the food into the air in the mouth and are delivered through swallowing and breathing to the olfactory epithelium in the upper part of the nose via the nasopharynx in what is referred to as the retronasal route [3]. Therefore, the concentration of retronasal aroma compounds at the olfactory receptors varies continuously with time, from the beginning of perception until fading, towards persistence and after-smell. Their release kinetics depends on the food matrix itself [7,11] but also on in-mouth physiological mechanisms, sometimes referred to as food oral processing, producing food breakdown [12,13]. Thus, salivation, mastication, and tongue movement conduct bolus formation with incorporated saliva and subsequent swallowing [12], mechanisms for which huge inter-individual variations exist due to physiological and behavioral differences [14,15,16,17,18]. Moreover, release gradients occur due to aroma compounds adsorption on the mucosa, inducing delayed releases that partly explain persistence and after-taste [3].

As temporal dimension is, therefore, of prime importance for any instrumental development aimed at studying in vivo aroma release, direct methods of analysis capable of continuously monitoring volatile compounds in the breath were an obvious option. Direct injection mass spectrometry (DIMS) techniques add this time dimension for the analysis of VOCs [19]. Thus, soft chemical ionization techniques using proton transfer, such as atmospheric pressure chemical ionization (APCI), proton-transfer reaction (PTR), or selected ion flow tube (SIFT) coupled to mass spectrometry (MS), are able to monitor in real time aroma compounds present in the exhaled breath directly in the nose [20]. Referred to as nosespace analyses [21], continuous breath-by-breath analyses [22] aimed at obtaining data that are supposed to reflect the pattern of aroma release in real time during food consumption, which is supposed to explicate perception [23]. These DIMS techniques accommodate the necessary instrumental constraints in terms of speed and response (linearity and limit of detection) and are compatible with real-time in vivo analysis of VOCs present in the breath of humans [20,24].

To account for dynamic and time-related aspects of aroma perception, specific time-dependent sensory methods have been developed [25]. Obviously, well-established static sensory measures such as descriptive analyses are not suitable. These measures made at a single time-point do not capture the full temporal sensory experience, and such evaluations are an integration over the time of the whole perception experience [26]. Very early, the time-intensity method (TI) that makes it possible to follow the intensity evolution of a sensory attribute was available [27]. Since TI focuses on a single attribute only, it is far from assessing the multi-component profile of aroma release and capturing the multidimensionality of perception. Therefore, other quantitative and qualitative temporal methods able to measure several attributes simultaneously have been proposed. Among quantitative-based ones, dual-attribute TI (DATI, [28]), modified TI [29], or multi-attribute TI (MATI, [30]) must be cited. Amongst qualitative-based ones, temporal dominance of sensations (TDS), which selects from a predefined list of descriptors which one is dominant at each time of consumption [31], and temporal check-all-that-apply (TCATA), which enables the selection of several pertinent descriptors at each time-point of consumption [32], are the most popular.

Combining dynamic instrumental and sensory methodologies in order to try to better understand aroma perception through the prism of aroma release seems desirable. Inter-individual variability in aroma release and perception, on the dependence of physiological and behavioral factors, is well documented, but intra-personal variations should also be taken into account. In time-dependent perceptive tasks, since continuous evaluation is highly attention demanding, individuals may vary in their sensory acuity and present fatigue detrimental to their capacity to stay focused on a particular task and the time elapsed between stimulation and response [8]. Therefore, important intra-individual variations are inherent to temporal sensory evaluation and may be related to circadian rhythm [8], mood, and physiological aspects like preprandial or postprandial status. Efficient training and planning tests at the same time in the day for each replicate session can reduce these variations in a certain extent [8]. However, aroma release experiments are also subject to intra-personal variations. Thus, although a strict breathing and sampling protocol was applied, significant intra-individual variabilities between replicates were effective in the nosespace analysis of flavored liquid samples, even if the five replicates were measured in the same session with sufficient recovery time between them [18]. The variation was proven to be due to human release behavior despite the strict evaluation protocol used; moreover, the variation was found to be volatile-compound-dependent, with a larger variation being obtained for compounds with a higher air–water partition coefficient [18]. Implication of intra-personal variations in aroma-release experiments could be an important issue for replicate measurements, particularly when replicates are being measured in different sessions at different moments. Aroma release and perception appear as multidimensional phenomena, with variations highly dependent on humans and with time-related aspects of inter- and intra-individual variability. As such, in order to understand better the mechanisms that link in vivo aroma release and dynamic sensory perception during food consumption, one should rely on a protocol where both instrumental and sensory methods are conducted simultaneously. Therefore, real-time simultaneous data capture seems preferable for measuring changes over time in order to avoid or at least reduce the inherent variability that would increase significantly if non-concurrent assessments are being used.

The aim of the present review is to present the temporal sensory methods available to analyze dynamically aroma perception and the in vivo dynamic instrumental methods used to analyze aroma release and to emphasize studies that conducted aroma-release and sensory evaluation simultaneously. Challenges related to various sources of variability, such as interactions with the food matrix, food oral processing, and sensory cross-modal interactions, are also discussed. Some perspectives for future research are also presented.

## 2. Dynamic Sensory Methods to Analyze Aroma Perception

To account for the dynamic character of flavor perception, temporal sensory methodologies that allow following the evolution of perceived sensations with time have been developed. The area of temporal methods has grown to such an extent that books (e.g., Hort et al., 2017 [25]) and reviews (e.g., Visalli and Galmarini, 2022 [33]) dedicated to the topic of time-dependent measures of perception in sensory evaluation have been published recently.

Historically, the first method proposed to follow the intensity evolution of a sensory attribute (e.g., one aroma descriptor) in the time course of food consumption was the time-intensity (TI) evaluation. Initially manually handled, data recording and treatment benefited early from computerized systems [27]. The TI evaluation had been considered as an established sensory methodology for some 40 years as early as in the beginning of the 1990s [34,35]. However, TI focuses only, although continuously and quantitatively, on a single sensory attribute. As a food consumption experience is rarely unidimensional, panelists must complete as many analyses as the number of sensory attributes to be evaluated. Quickly, the methodology can become lengthy and costly. Moreover, continuous TI, sometimes referred to as CTI [36], requires extensive training of the panelists who tend to reproduce a stereotypical response. It is also subject to the halo-dumping effect [8,37], when intensity evaluation of one descriptor can be biased by the concomitant perception and influence of other attributes, questioning the quantitative measure [36,37]. To tackle this problem, a method that allows evaluating the intensity of two attributes simultaneously has been proposed (dual-attribute TI or DATI, [28,38]). Although DATI tests require half the time to complete in comparison to successive single-attribute tests [28], they does not fully answer to the multidimensionality of flavor perception and need careful panelists’ training but are particularly suited to study the relationships between two attributes (e.g., one taste and one aroma, [28]). The data parameters generally used with TI curves are the maximum intensity (Imax), the time to reach Imax (Tmax), and the area under the curve (AUC) that measures the overall perception.

To examine the halo-dumping effect, Clark and Lawless [37] developed a discontinuous TI methodology using specified discrete time-points to simultaneously follow two flavor attributes (i.e., sweetness and aroma). They found that odor-induced enhancement of sweetness was effectively lessened or eliminated [37]. Discrete (discontinuous) time intensity (so-called DTI), used to evaluate perceived intensities at specified, distinct time-points [39], involves the rating of single or multiple sensory attributes at these discrete points in time [39]. DTI can measure temporal changes over longer periods and appears more flexible while allowing for more than one attribute to be assessed at once, lessening or eliminating the attribute-dumping effect. However, its discrete nature means that some information may be lost between time-points, data are often noisy, and the design of experimental protocols could be rather complex [40]. It does not appear as a method of choice for assessing changes over short time periods [40] but seems to better suit long evaluation periods, such as persistence or after-taste phenomena. However, this was the basis of a so-called “modified TI” method developed to evaluate the time-related intensities of various flavor descriptors of a model cheese consumed within one bite [29]. Schematically, the attributes were presented to each panelist alternatively and randomly during successive masticatory sequences of ca. 3 min. During these periods, which were divided in nine measuring time-points, the descriptors were randomly presented on a computer screen at each measuring time. The assessors had to evaluate their intensities at each time-point during a 3 s delay. Therefore, a complete evaluation of several descriptors necessitated several sequences for each panelist [29]. The method, later rationalized as the multi-attribute TI (MATI) method [30,39], although avoiding the attribute-dumping effects, keeps the drawback of a lengthy and potentially costly method. In fact, an extended time duration is required to cycle through the attributes list repeatedly in order to capture a sufficient number of points to model the MATI curves [39]. Moreover, as papers using these techniques are particularly scarce, concern has been raised about the difficulty for participants in handling the procedure [35]. A derived method called alternate time intensity (ATI) has been used recently to evaluate two sensory modalities, namely the salty taste and the specific aroma of flan [41], but it appears quite equivalent to the DATI, “modified TI”, or DTI methods described above.

TI was initially considered as a kind of temporal version of quantitative descriptive analysis (QDA) while allowing measuring only one attribute at a time [33]. However, descriptive analyses, with the evaluation of the intensity of several descriptors that gives rise to common sensory profiles of foodstuffs, are static methods. The descriptors intensities are integrated by the panelists at one time, which can be different for each panelist, all over a sensory session. Some discontinuous temporal versions of descriptive analyses that somewhat simplify the procedure [33] have been published, such as intensity variation descriptive methodology (IVDM, [42]), an early version of progressive profiling (PP, [43]). They enable the measure of attributes intensities within a single intake at uniform interval steps or at specific moments and allow quantitative profiles of several attributes at different times to be obtained. Time scanning descriptive analysis (TSDA) was originally designed to tackle perception discrepancies caused by differences in evaluation temperatures between panelists evaluating hot beverages [44]. As such, as an alternative to QDA, TSDA accounts for the dynamics of sensory perception as a function of the temperature of the test sample at which it is evaluated [44]. Nevertheless, it could also be used as a convenient dynamic descriptive method, as it introduces intensity scaling of specialty attributes at designed time blocks. Based on the same time-steps evaluation process but for multiple intakes during consecutive consumption protocols, sequential profiling (SP), which extends progressive profiling to multi-bites (or sips), has been proposed [45]. Finally, a very demanding dynamic flavor profile method combining the descriptive approach and the TI approach has been described [9]. It consists of recording the TI response of each descriptor previously determined by QDA; a 3D drawing of the data allows obtaining a descriptive profile of a food at each consumption moment [9]. The quantitative methods described above require the generation of the attributes by the panel prior to the main analysis, which requires anterior descriptive tasks. They are all demanding methods that share the requirement of highly trained assessors and the drawback of a quite lengthy (and costly) process, which almost precludes simultaneous nosespace analyses if several sensory attributes are in question.

**Figure 1:**
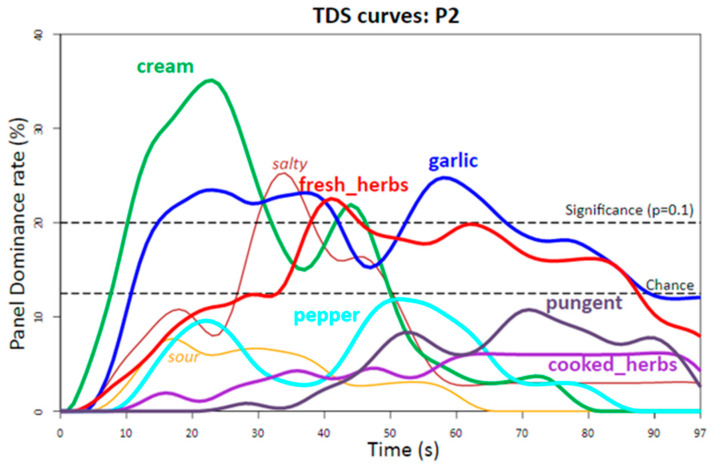
TDS curves obtained for a flavored (“garlic and herbs”) fresh cheese (P2) evaluated by sixteen panelists using eight predefined attributes: garlic, cream, fresh herbs, cooked herbs, pungent, pepper, salty, and sour [48]. On these graphs, the “chance level” corresponds to the dominance rate that could be reached by chance for a given attribute. Its value, P0, is equal to 1/*n*, with *n* being the number of attributes. The “significance level” is the minimum value that must be reached for the dominance rate to be considered significantly higher than P0 and is calculated from the confidence interval of a binomial proportion based on a normal approximation [31]. Only TDS curves above the significance level are considered significantly dominant.

In order to simplify the task, qualitative dynamic methods have developed recently. Temporal dominance of sensations (TDS, [31]) is a method that allows evaluating dynamically several sensory attributes (up to twelve in practice [46]) simultaneously during consumption. Methodologically, TDS lies between conventional static descriptive analysis of several descriptors and dynamic but unidimensional TI [35,46]. The method has developed considerably, including data treatment advances [47], and could be considered as the ideal means to dynamically follow the sensory perceptions associated with the release of aroma-active molecules. However, TDS introduced the concept of dominance, which is different from intensity. Practically, the panelists have to continuously identify during consumption the dominant sensation among a given list of attributes consensually determined beforehand. Therefore, a TDS analysis results in a sequence of dominant sensations measured by the dominance rate of the panel (Figure 1) during the evaluation period that can include a post-ingestion time to evaluate persistence [46].

However, the dominance notion is a complex mental construct that includes more than one single aspect of sensory perception [49]. With TDS, only the most salient sensations at a given time are dynamically assessed reliably. Therefore, some descriptors, although being important to explain particular perceived sensations whose intensities could be possibly evaluated in a descriptive analysis, may appear as not noticeable because they are never significantly dominant (e.g., pepper, pungent, and cooked herbs aromas in Figure 1).

**Figure 2:**
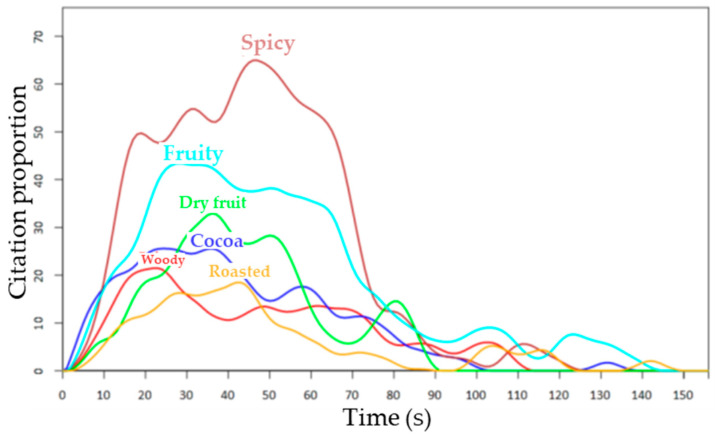
TCATA curves obtained for a dark chocolate evaluated by sixteen panelists using six predefined aroma descriptors [53]. Results are expressed as panel citation proportion.

As an alternative to TDS, the temporal check-all-that-apply method (TCATA, [32]) was developed. TCATA integrates the temporal monitoring of all applicable attributes chosen among a predefined given list and transforms the static CATA (check-all-that-apply) method [50] into a dynamic one. Method and data treatment developments have made TCATA as operational as TDS [51,52]. Allowing a dynamic monitoring of several attributes (and not only the dominant ones), TCATA seems attractive (Figure 2) for hyphenation to aroma-release analyses.

However, as for TDS, some drawbacks soon appear in practice: a limited number of attributes in the predefined list and difficulties in handling the method for the panelists. (They have to check descriptors when they are perceived and uncheck them when they are no longer apparent.) However, a familiarization step seems to afford a certain comfort improvement [51]. Moreover, for both methods, data treatment averages results on the panel, leading to the loss of individual information.

The two methods were compared very early [54], and both were found equally adequate to discriminate food samples [54]. TCATA was found slightly more efficient, as it gives rise to more detailed dynamic sensory profiles [54,55]. However, this alleged preponderance could depend on the type of food samples, on panels performance (use of familiarized, trained, or naïve panels), or on the lack of specific criteria necessary to compare the two temporal methods [47]. TDS suffers from dithering and dumping effects, which can be important when two or more sensory modalities are being evaluated in the same sequence (e.g., aroma and/or taste and/or texture). Then, only a few attributes are available for each modality, and the panelists can be indecisive when choosing modality and descriptor at the same time [49]. To alleviate this drawback, TDS by modality (M-TDS), which differentiates TDS sequences for each sensory modality, was proposed [56,57], with, however, the inherent disadvantage for the panelists to perform as many evaluation sessions as modalities to be evaluated. The three methods (TDS, TCATA, and M-TDS) were compared in a study aimed at characterizing semi-solid foods of yogurt type [58]. They were found equally able to discriminate the samples, with some advantages for M-TDS and TCATA for the dynamic multimodal sensory description of the products. TCATA afforded additional information on potential interactions between modalities or/and descriptors. The three methods seem adequate for the discrimination of a set of samples, but they have never been compared in a combination of aroma-release analyses in order to explain perception. Moreover, this comparative-only study was conducted using a single, intensively trained panel in a predetermined evaluation order (TDS, TCATA, and then M-TDS), and no definitive conclusion on the respective advantages and drawbacks of the three methods could be inferred [58]. The use of trained panels or naïve consumers for these qualitative temporal methods is also debated [58]. In a recent study on chocolate-hazelnut spreads consumed without or with a carrier food by a consumer panel (*n* = 72), TCATA better discriminated between spreads, while TDS revealed clearer temporality of sensations [59].

In the method presented above, the studied time-lapse mostly concerns the time it takes a subject to continuously evaluate one intake (sip or bite) of a food product from the moment of food intake in the mouth until a few moments after swallowing. However, food and beverage consumption generally needs multi-intake, and the temporality of full-portion evaluation (bite after bite or sip after sip) has been rarely studied [33]. Thus, with the objective of measuring the order in which key attributes appear over a complete eating experience (including each mouthful until aftertaste), temporal order of sensations (TOS) was proposed [60]. Within a predefined list of attributes, the subjects were asked to indicate for each bite/sip and for aftertaste in order which three, for instance, they perceived first [40,60]. The results were then presented according to the proportion of each attribute emerging first at each evaluation time. Another method, the so-called “pick 3 and rank” (P3R, [61] cited by [62]), was proposed to measure the temporality between several bites of a full portion. P3R consisted of retrospectively (i.e., not in real time) picking and then ranking the three most important descriptors perceived during a bite [62]. Based on TOS and P3R, a new retrospective temporal method called attack-evolution-finish (AEF), inspired from the sequence often used by wine professionals [62], was recently proposed [62]. It consists of retrospectively selecting the most important descriptor during each of the three tasting periods [62], with this selection being related to the concept of dominance rated in TDS [62]. In a comparison in a study on dark chocolates, it was concluded that AEF and TDS produced very similar results in terms of product discrimination [62]. In order to avoid the potential bias induced by presenting a limited number of predefined descriptors to the panelists, it was proposed recently to use free comments with AEF (FC-AEF, [63]). FC-AEF allows the subjects to evaluate their temporal perception using their own words instead of a limited predefined list of attributes. Used with a panel of 63 consumers evaluating five dark chocolates, thus avoiding the necessary training of assessors, it was claimed that FC-AEF was able to provide temporal discrimination and characterization of the products [63].

The newest methods escaped from the real-time simultaneous tasting-evaluating paradigm, as they propose a retrospective evaluation of several attributes at discrete moments of consumption immediately after tasting [33]. Advantages and disadvantages of concurrent versus retrospective sensory data collection are discussed in detail in recent papers that proposed the retrospective measures [62,63,64]. While losing temporal resolution in real time, it is claimed that retrospective methods do not require training or familiarization and, therefore, can be implemented without difficulty with naive consumers compared to other temporal methods [64]. They seem adequate for discriminating food samples. However, they still need to be confronted with dynamic instrumental aroma-release methods in order to evaluate their ability to strengthen the release–perception relationship issues.

## 3. Dynamic Instrumental Methods to Analyze In Vivo Aroma Release

Aroma compounds directly measured in the expired air from the nose (so-called nosespace) during food consumption are supposed to reflect aroma release in real time. They are supposed to be representative of the molecules that interact with the olfactive receptors (the active odorants) via the retronasal route, hence causing aroma perception.

Techniques for measuring the aroma released in air expired from the human nose have been developed during the last three decades. Significant, robust results were obtained for sampling aroma using a collection of expired air samples at discrete time-points during consumption on adsorbents of Tenax^®^ type [65,66] or on solid-phase microextraction (SPME) fibers [67,68]. After thermal desorption of the adsorbent and the analysis by gas chromatography coupled to mass spectrometry (GC-MS) of each desorbed sample, it was possible to construct aroma-release curves although obtained discontinuously (e.g., Linforth et al., 1996 [69] and Pionnier et al., 2004 [67]). Moreover, this discontinuous sampling presents a low sensitivity due to the limited adsorption time available for each time-point. Therefore, this method is essentially used to study highly flavored model foods. Real-time continuous in vivo aroma analysis has been obtained using atmospheric or sub-atmospheric pressure ionization MS [70], often referred to as direct injection MS (DIMS, [19]). Aroma-release curves are thus obtained in real time in a continuous way, as air from the nose is sampled directly into a mass spectrometer through a heated interface, making real-time breath-by-breath analysis routinely possible. These techniques operate in soft chemical ionization (CI) mode [24], generally by proton transfer from the reactant hydronium ion H_3_O^+^. Most of the volatile compounds have higher proton affinities (PA) than water (PAH_2_O = 691 kJ/mol), and they ionize by proton transfer from H_3_O^+^, giving rise essentially to protonated molecular ions MH^+^ and a few fragments [70] that are accelerated into a mass spectrometer. Advantageously, common constituents of air have PAs lower than the PA of water and are not ionized. Among the DIMS techniques, those that are based on proton-transfer CI, such as atmospheric pressure chemical ionization (APCI-MS), proton-transfer reaction (PTR-MS), and more recently, selected ion flow tube (SIFT-MS), appear especially well suited to explore the dynamic process of aroma release [19,20,70], and they have been used quite extensively for the last twenty years. Secondary electrospray ionization (SESI-MS) has demonstrated some potentialities in the domain of real-time breath analysis (e.g., Berchtold et al., 2014 [71], Gaugg et al., 2016 [72], Weber et al., 2023 [73]) and should also contribute to real-time aroma-release studies in the near future. Noteworthy, the technique has recently proven its utility in VOCs fingerprinting [74]. These DIMS techniques accommodate the necessary instrumental constraints in terms of fragmentation, speed, and response (linearity and limit of detection) and are compatible with in vivo analysis of volatiles present in the breath of human subjects [75]. From the early beginning, many examples of nosespace analyses and their fundamental advances may be found in dedicated or specialized treatises [24,75,76,77,78,79].

The first significant, robust results were obtained using APCI-MS with an ion source optimized for the detection of volatile substances hyphenated to a quadrupole mass analyzer [69,80]. This so-called MSNose™ interface [81] has been used in numerous aroma-release studies that can be found in dedicated reviews [22,23,79,82]. Aroma-release curves also reflect the respiratory cycle of individuals (signal increase on exhalation and signal decrease on inhalation), allowing the measure of respiratory frequencies. From the start, inter-individual differences in aroma-release kinetics, linked to oral physiology variability, were evidenced [80] and compound-dependent temporal release delays, linked to in-mouth enzymatic reactions, disclosed [69]. Optimized APCI sources have also been interfaced with ion-trap mass spectrometers [75,83,84,85,86] or triple-quadrupoles [87], providing sensitivity, selectivity, and structural capability benefits of tandem MS (MS/MS). Instruments sensitivity is an important issue, as only a minor part of the food aroma components is actually able to reach the nasal cavity [3]. A large part of the odorants is simply swallowed with the food and adsorbed via the gastro-intestinal tract [88]. Moreover, in the mouth, odorants partition between food media and saliva or air, adsorb on the mucosa, and are diluted in the breath during transport to the nose. All these compound-dependent events limit their availability for perception [12]. Thus, retronasal concentration has been reported to reach only 0.1 to 10% of the concentration in the food measured by static headspace and to be 10 to 100-fold lower than in the mouth [89]. APCI instruments hyphenated to quadrupole mass analyzers present some limitations. To reach the sufficient time resolution and sensitivity necessary for in vivo aroma-release studies, they need to be run in the multiple-ion detection mode (or selected ions monitoring) with dwell times that limit the practical measurable number of targeted ions (i.e., individual VOCs) to 5–10. This limitation is also true for PTR-MS and SIFT-MS instruments run with quadrupole analyzers. Contrarily, ion-trap instruments are fast analyzers that allow full scans to be performed and are compatible with the necessary time resolution of in vivo aroma release, with comparable sensitivity to that of multiple-ion monitoring of quadrupole instruments. Therefore, ion-trap mass analyzers allow untargeted analyses. Numerous examples of in vivo nosespace studies using APCI-MS may be found in dedicated publications (e.g., [19,24,75,79,82]). The PTR-MS technique [90,91,92] has been also largely used contemporarily to APCI for real-time breath and nosespace analyses [18,93,94]. Contrarily to APCI, where reagent ions are produced in the vicinity of the ionization region in the source, in PTR, the generation of the reactant H_3_O^+^ ion in a specially designed source is spatially and temporally separated from the proton-transfer reaction that occurs in a dedicated reaction chamber, the drift tube [20]. Therefore, a better control of the ionization process is achieved, and individual optimization and quantitation are made accessible [20,95]. The same applies to the SIFT-MS technique [96], where the reaction chamber is a flow tube, with the additional advantage of thermal energy ionization conditions that allow studies on reaction rate coefficients between reagent ions and neutral analytes [20]. However, the necessary addition of a carrier gas in the flow tube produces a dilution of the breath samples, thus limiting de facto the sensitivity of the technique. Used for a long time in breath research [97,98], SIFT-MS has naturally proven its utility in nosespace analyses [99,100,101,102,103].

A major breakthrough was achieved when a time-of-flight (ToF) mass analyzer was hyphenated to PTR instruments [104,105]. As already outlined, quadrupole mass analyzers (QMA) are limited to analyses targeted to a few analytes whose molecular ions or fragment ions are obtained at nominal masses. ToF affords higher mass-resolving power that allows isobaric compounds to be distinguished, greater speed of analysis in full scan mode, and a better sensitivity on the whole mass range. These features afford tremendous benefits, with full scans that are compatible with breath-by-breath time-resolution and sensitive, untargeted analyses. While nosespace analyses conducted using QMA generally address highly flavored food models or foodstuffs reinforced with a few aroma molecules, ToF instruments may address real food issues [106]. Moreover, tentative identifications are made possible with theoretical assignment of sum formulae to each detected ion thanks to the exact mass measurement allowed by the mass accuracy of the ToF analyzer. However, a sum formula may correspond to one or more compounds (isomers), and it remains advisable to ascertain aroma composition by GC-MS to provide confirmatory data on compound identities [20]. Numerous examples of nosespace analysis using the PTR-ToF-MS technique can be found in recent publications [24,77,107]. It is noteworthy that both SIFT-MS and PTR-MS ion sources have the possibility to produce other reactant ions such as O_2_^+.^ and NO^+^ that ionize neutral molecules via charge transfer or hydride abstraction. As an intrinsic feature of SIFT-MS in the “selected ion” process (negative reagent ions O^−^, O_2_^−^, OH^−^, and NO_2_^−^ may also be selected), it necessitates a switchable reagent ion (SRI) option for PTR-MS [108]. The ammonium ion NH_4_^+^ may also be used as a protonating agent using both instruments [109,110]. Although all these reagent ions can be useful in specific applications (e.g., OH^−^ for detecting molecules bearing labile protons or NH_4_^+^ for detecting amines and labile oxygenated compounds), they still need to demonstrate their usefulness in routine aroma-release analyses.

**Figure 3:**
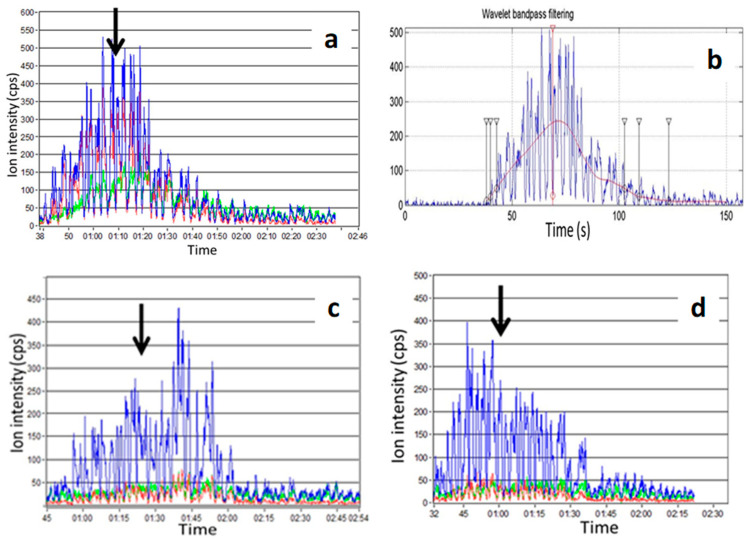
Examples of aroma-release curves obtained for a flavored (“garlic and herbs”) fresh cheese evaluated by sixteen panelists using a PTR-ToF-MS (in blue, *m*/*z* 73.067 butan-2-one; in red, *m*/*z* 87.044 diacetyl; in green, *m*/*z* 89.060 butyric acid/acetoin) [48]. Vertical arrows on (**a**,**c**,**d**) stand for swallowing time. The release curves illustrate inter- and intra-individual variability: (**a**) stands for one panelist, while (**c**,**d**) are replicates of another panelist. Vignette (**b**) displays an example of signal smoothing (red trace is the smoothed signal of *m*/*z* 73.067) of (**a**) using wavelet bandpass filtering [111].

As for the data treatment used in TI, the data treatment parameters generally retained for the release curves are the signal maximum intensity (Imax), the time to reach this maximum intensity (Tmax), and the area under the curve (AUC) that measures the total amount of the released compound. The curves may be smoothed to attenuate the up and down signal variations due to breathing (signal up on exhaling and signal down on inhaling), and thus determine more easily the Imax and Tmax parameters (Figure 3).

## 4. Aroma-Release and Sensory Evaluation Conducted Simultaneously

Many attempts to relate in vivo dynamic aroma release to perception relied on static sensory methodologies such as descriptive analysis. Both analyses were conducted at different moments and not simultaneously for practical impossibility. Sometimes conducted using the same panel for both analyses, a majority of studies employed a different panel for sensory evaluation, often with more panelists for more robust sensory results. Static sensory methods, which result in an integration by the panelists of each sensory modality, are not able to assess time-related aspects of perception. Moreover, as the panelists meet together at a different moment, individual aspects apprehended by nosespace analyses, which are compulsorily conducted at the individual level, are lost. In efforts to establish the link between release and perception, not taking into account those inter-individual variations may result in possible bias, and static sensory methods are essentially good at differentiating products. Nevertheless, some significant relationships have been established on the effect of fat, thickener, or sugar on aroma release and perception in a descriptive approach. For instance, a descriptive profile was used in a study on the effect of thickener type and fat concentration in citrus emulsions [112]. Thus, it was found that delivery to the nasal cavity of the most lipophilic compound was affected by fat, and both fat and thickeners influenced perceived flavor and texture, which could be explained by instrumental data in most cases [112]. Moreover, a protocol based on descriptive sensory analysis was proposed to study flavor release and perception in cheese [113].

Observed discrepancies between release and perception were hypothesized to be due to interactions not taken into account by static methods. Dynamic sensory methods (essentially TI) have also been used in parallel while independently of the release studies, either by the same panel or by two different panels. While, as previously stated, inter- and intra-individual variations may be responsible for some bias, significant results have been obtained. Thus, for instance, multi-attribute TI allowed establishing some relationships between sensory attributes and released flavor compounds in a model cheese [29], and discontinuous TI was used to decipher the respective role of aroma release, salivary composition, and oral processing parameters in model cheese aroma perception [114].

Therefore, different strategies have been adopted to investigate non-concurrently the dynamic aspects of aroma release and aroma perception during food consumption. Conducted essentially on model foods using static or dynamic sensory methodologies by single or different panels, observed discrepancies in the results have been often linked to the complexity of the involved phenomena, including matrix effects, cross-modal interactions, and inter-individual variations. Therefore, although interesting information has been obtained in parallel studies, as already outlined, to clearly establish the link between in vivo aroma release in the nosespace and aroma perception and to avoid bias as much as possible, it seems advisable to conduct the two complementary analyses simultaneously, with the additional advantage of single-panel appropriateness. Table 1 groups the concurrent studies conducted so far on model foods, real foods, and composite foods, which have been generally flavored for easier interpretable results. Emphases on types of measured effects and on methodological developments are presented below.

Due essentially to limited sensitivity inherent to APCI- or PTR-quadrupole instruments for in vivo studies, real food systems were rarely investigated initially. Thus, the first studies conducted simultaneously dealt with highly flavored model food or flavored-reinforced real food (Table 1) while staying sensorially acceptable for the panelists.

Using a gelatin gel flavored with increasing concentration of carvone (Table 1, [115]) or a solid food aromatized with a rosemary flavor (Table 1, [5]), a perfect correlation between aroma release and simultaneous perception was found (Table 1). In the carvone experiment, the aroma quantity delivered in the nosespace was proportional to the carvone concentration in the product, although absolute measured quantity varied greatly between individuals, and the perceived mint intensity was correlated to the aroma concentration [115]. These simple models seem to attest to a clear link between release and perception. The time of maximum perceived intensity (Tmax of TI experiment) occurred before the time of maximum aroma release (Tmax of nosespace experiment), and this was attributed to adaptation [116] to the stimulus [115]. Huge inter-individual variations were noticed, but the speed of eating seemed to influence the level of adaptation to the stimulus; the slower the eating event, the greater the adaptation [115], confirming previous results obtained by Linforth et al. with more complex models (Table 1, [117]).

Aroma molecules are generally hydrophobic entities, and therefore, food fat content is an important factor that affects aroma release and perception. The link was investigated in flavored yogurts varying in fat content [118]. Nosespace measurements revealed faster and more intense aroma delivery but with less persistence in low-fat yogurts (Table 1). Sensory data also revealed faster and more intense aroma perception in low-fat samples, while no significant differences were found between samples for perceived persistence [118]. Similar results were obtained with flavored milks varying in fat content (Table 1, [119]) and with flavored liquid emulsions differing in fat level (Table 1, [120]). An adjustment of aroma content in low- and high-fat milks in order to deliver the same aroma quantity resulted in isointense aroma perception [119]. In these examples, the effect of fat on in vivo release globally conformed to the theory when significant decreases in both in-nose volatile release and corresponding perceived aroma intensities were found on increasing fat content. However, contrasted results were observed when considering the respective hydrophobicity of the aroma molecules (Table 1, [118]), which was later confirmed by an experiment conducted non-concurrently on flavored milk with varying fat content [121]. In flavored emulsions, the fat content also produced a significant effect on pre- and post-swallowing events [120]. With a rigorous breath and consumption protocol, it was found that on increasing the fat content, the ratio of volatiles released in the post-swallow phase increased significantly compared to the pre-swallow phase. The authors hypothesized that these data could contribute to understanding why low-fat and high-fat foods are perceived differently [120]. These results globally confirmed others that were obtained in non-concurrent analytical/sensory experiments (e.g., [122]). However, changing fat content in food may affect other parameters such as structure and texture with, for instance, differences in particle size and viscosity, which could also affect aroma release [118]. These structure and concomitant texture issues have been the subject of several studies on the impact of various thickeners on aroma release and perception (Table 1).

When the only studied parameter was the thickener (e.g., gelatin or whey protein) concentration, in mono-flavored model gels, it was clearly established that texture determines aroma perception rather than in-nose aroma concentration, evidencing texture–aroma cross-modal interactions (Table 1, [17,123,124]). Moreover, perception seemed correlated to the different rates of aroma release in differently textured gels [123]. Quick release during the chewing phase was noticed for soft gels, while aroma delivery from hard gels increased slowly, reaching maximum intensity during swallowing [17]. However, for soft gels, individual release curves presented two different patterns. Some panelists released aroma during the chewing phase, while others released aroma essentially on swallowing. A proposed explanation was that the latter kept their velum (soft palate) unconsciously closed during jaw movement, while the former opened their velum, allowing aroma transfer to the nose, already changing their chewing behavior according to that of a more rigid system [17]. Directly related to aroma delivery, aroma perception showed high temporal resolution largely modulated by individual physiological differences that were not fully mirrored by the TI sensory methodology [17].

TDS was applied to differently textured gelified candies (Table 1) flavored with three aroma molecules imparting “green”, “strawberry”, and “butter” olfactory notes to the candies (namely Z-hex-3-en-1-ol, ethyl hexanoate, and diacetyl, respectively). TDS sensory data indicated a linear increase in the global duration of dominance in hard candies [125]. An imposed in-mouth melting protocol revealed interesting features. Temporal sensory profiles were found to be dependent on the candies’ texture. Thus, the “strawberry” attribute was dominant in the liquid gel (0% gelatin). In the 2% gelatin gel, the “green” attribute dominated before the “strawberry” descriptor became significantly dominant. For the 5% and 15% gelatin candies, “butter” was dominant in the initial melting time before “strawberry” became dominant in perfect coincidence with swallowing [125]. Temporal relationships with corresponding released aroma molecules in the nosespace were evidenced. When the panelists chewed the candies freely, out of the melting protocol, quicker (Tmax) and a higher amount (Imax) of aroma release was obtained, but only the “strawberry” attribute was judged as dominant around a shortened swallowing time. In this case, temporal relationships between release and perception were more difficult to establish. The use of an imposed melting protocol involving a longer residence time of the product in mouth probably allowed easier relationships to be evidenced. However, all the conclusions were mainly based on a descriptive analysis of instrumental and sensory data averaged on the panel, where the variety and the complexity of the phenomena could not be fully apprehended [125].

When a mechanical treatment was coupled to varying protein content in flavored, stirred yogurts in order to obtain varying yogurt viscosity, the different viscosities influenced aroma release and perception (Table 1, [126]). However, the phenomena were more influenced by the applied mechanical treatment than by protein content, revealing the importance of viscosity in the release and perception relationships [126]. Finally, when differing sugar levels were combined with various thickener contents in flavored model custards, perceptual sweetness–aroma cross-modal interactions were mainly evidenced, whatever the texture of the desserts (Table 1, [127]).

One of the first clear demonstrations of a sweetness–aroma cross-modal interaction was obtained using mint-flavored chewing gums in a combined nosespace–TI experiment (Table 1, [128]). If menthone release and mint perception concomitantly increased quickly, mint perception rapidly decreased, while the nosespace menthone content remained almost constant. It was found that mint perception followed the decrease in the sucrose level measured in the saliva of the panelists, hence the sweetness perception. Thus, a taste/aroma interaction between sweetness and the congruent mint aroma was evidenced [128]. However, although the panel was rigorously trained for aroma TI assessments, one cannot exclude that the assessors might have confused loss of sweetness with mint flavor [128]. Alternatively, the panelists could have become adapted to menthone with time, and this adaptation [116] period could have coincided with the sucrose-release time [128]. To limit adaptation, the food matrix must have a shorter residence time in mouth. This is clearly the case for beverages. The effects of sugar and CO_2_ levels were investigated in mint-flavored (menthol, menthone, and (Z)-hex-3-en-1-ol), carbonated beverages in the context of reducing sugar levels in such beverages, which represents a challenge for the soft drink industry (Table 1, [129]). While increasing CO_2_ levels resulted in increased aroma release and perception regardless of sucrose content, increasing sugar concentrations only tended (not significantly) to induce higher aroma release in the nasal cavity, with, however, no effect on perception for carbonated beverages [129]. Perceptual sweetness–aroma interactions between congruent flavors (mint and sweet) were thus evidenced in the absence of CO_2_. Similarly, Lethuaut et al. found that a higher level of sucrose in their flavored gels increased aroma perception without a significant effect on aroma release (Table 1, [127]). The presence of CO_2_ in the carbonated beverages could have induced a trigeminal perception that masked aroma perception in a perceptual trigeminal–aroma interaction or could have increased complexity, causing difficulties with assessing the carbonated products [129].

Real foods have also been investigated with simultaneous instrumental/sensory protocols. Thus, three commercial soft cheeses were submitted to nosespace (APCI-MS) and dynamic sensory (TI on the three predefined “sulfury”, “buttery”, and “mushroom” attributes, evaluated in separate sessions) analyses (Table 1, [130]). This study revealed the sensitivity and technical limits of APCI-QMA instruments for studying real foods. While 19 compounds were identified as major odor-active volatiles of the cheeses by GC-O, only 6 protonated molecular ions were detected in the nosespace of 15 panelists, namely *m*/*z* 63 (dimethylsulfide), *m*/*z* 91 (S-methylthioacetate), *m*/*z* 94 (dimethyldisulfide), *m*/*z* 87 (3-methylbutanal/diacetyl), *m*/*z* 115 (heptan-2-one), and *m*/*z* 143 (nonan-2-one). Temporal correlations were established only between the “sulfury” note and the three sulfur compounds. Poor correlations displayed for the other attributes were mainly due to insufficient sensitivity of the APCI-MS for some key odorants (octan-3-one and oct-1-en-3-ol, responsible for the “mushroom” note, were not detected in the nosespace) and unachieved separation of isobaric compounds (diacetyl, responsible for the “buttery” note and found in low concentration, and 3-methylbutanal, which was present in much higher concentration) [130].

Two protocols (spitting out and swallowing) were compared for the evaluation of aroma release and perception of an alcoholic beverage (a commercial flavored vodka) using TDS as the sensory methodology (Table 1, [131]). Significant differences in both release and perception were observed when comparing the two protocols. The more usual swallowing one produced successive dominances that were more complex but decreased the panel’s dominance rates of the significant attributes. Ethanol perception was also important when the beverage was swallowed. Aroma-release data accounted only partly for the perception differences. Nevertheless, despite the lack of specific key odorants of the predefined sensory attributes, some temporal parameters of release data (mean values on the panel) could be related to the time at which dominance appeared and to the dominance duration of some attributes. However, only limited conclusions could be inferred due to the variety and complexity of the involved mechanisms [131]. On the one hand, all aroma compounds fragmented to a large extent under the chosen PTR-MS operating conditions, and most of the fragments were common to several molecules; targeted analysis imposed by the quadrupole mass analyzer allowed monitoring a limited number of ions representing several volatiles. On the other hand, as already outlined, TDS revealed only the significant dominant sensations at panel level, and mean values of the release parameters were used for a descriptive-only linking of analytical and sensory data [131].

As outlined above, the launch of the PTR-ToF-MS technology changed the game for real food systems. In a study on espresso coffees, a roasting effect was evidenced by both TDS and nosespace data obtained concurrently (Table 1, [132]). Thus, a change in aroma perception was observed when roasting increased, with a switch of dominance from “roasted” to “burnt”. At the same time, more volatiles in higher concentrations were released in the nosespace with increasing roasting degree [132]. Moreover, TDS revealed differences in aroma dominances between samples at the middle/end of perception time, while the release of potent odorants displayed different behaviors. Thus, untargeted volatile tracers of the “burnt” sensory attribute could be tentatively identified as *N*-heterocycles, such as substituted methyl-pyrroles and pyridine [132]. Sugar addition did not modify the nosespace composition but completely altered coffee perception. As expected, the “sweetness” attribute became dominant, and an increase in the perceived aromatic complexity with time was evidenced, with “caramel” and “hazelnut” gradually dominating the “roasted” and “burnt” notes. Congruency between sweet taste and several coffee aromas thus provoked a perceptive taste–aroma cross-modal interaction that could be temporally monitored by both dynamic methods [132]. Furthermore, a cluster analysis conducted on the nosespace data allowed characterizing two different temporal behaviors for aroma release, making possible the identification of potential markers of the temporal dominances revealed in TDS [132].

Relationships between release and perception have been obtained essentially thanks to temporal links obtained through descriptive analyses of the two types of data averaged at panel level. However, intra- and inter-individual behavioral variations in both release and perception have been evidenced, which complicates interpretation and prohibits a complete understanding. Taking into account those differences in data treatment could improve our knowledge of such mechanisms, which are recognized as particularly complex. In an attempt to better correlate the two types of data, it was proposed to use an index calculated at an individual level for each panelist and replicate in a study on commercial flavored (“garlic and herbs”) fresh cheeses that paired nosespace PTR-ToF-MS and TDS (Table 1, [48]). This index was defined as the abundance of each compound detected in the nosespace of each panelist while a given attribute was dominant; it was called AWD, i.e., abundance while dominance. As this index was computed at the individual level, statistical treatments were made possible, and correspondence analysis (CA) of the resulting AWD contingency tables was suggested to highlight the potential relationships between the aroma compounds released in the nosespace and the sensory attributes [48]. Among all the ions detected in the nosespace using PTR-ToF-MS, sixteen aroma compounds characterized by the experimental exact mass of diagnostic ions postulated to be their protonated molecular ions MH^+^, whose identities were confirmed by independent GC-MS analyses of the products, were selected. They were characteristic of dairy aroma, with additional sulfur compounds probably coming from garlic and terpenes probably coming from herbs. Their MH^+^ masses ranged from *m*/*z* 61.028 (acetic acid) to *m*/*z* 179.002 (diallyltrisulfide). Meanwhile, eight predefined sensory attributes were followed using TDS: “garlic”, “cream”, “fresh herbs”, “cooked herbs”, “pungent”, “pepper”, “salty”, and “sour”. Averaged over the entire panel, the release curves showed a clear temporal effect with significantly longer Tmax for some molecules. Thus, diacetyl (*m*/*z* 87.044) appeared early, followed by terpenes (*m*/*z* 137.132) and sulfur compounds (like diallyldisulfide at *m*/*z* 147.030). These could correspond to the successive panel dominance rates of the “cream”, “garlic”, and “fresh herbs” attributes that reached significance [48]. Other molecules were released later, such as 3-vinyl-1,2-dithi-4-ene (*m*/*z* 145.017), whose Tmax was delayed by more than 30 s compared to diacetyl Tmax. The latter, together with butyric acid (*m*/*z* 89.060), also lasted longer in the breath, and both could correspond to the long-lasting “pungent” sensation significantly dominant at the end of the evaluation sequence. The CA of the contingency tables containing AWD mean scores for each product revealed significant correspondences between attributes and ions. Thus, the “cream” attribute was clearly associated with diacetyl and butan-2-one. The “pungent” and “pepper” characteristics were associated with carboxylic acids but also, to a lesser extent, with the sulfur compounds 3-vinyl-1,2-dithi-4-ene and diallyltrisulfide. The “garlic” sensation was clearly associated with a mixture of sulfur compounds, and the herbaceous-related attributes seemed associated with a mixture of odorants. These encouraging results confirmed the tendencies found in the simple descriptive associations based on temporal considerations while extending the possible correspondences between attributes and ions. However, the authors concluded on the necessity of developing analyses that are more sophisticated to statistically assess the significance of the relationships between multiple key-aroma compounds released in the nosespace and the temporal perception of real foods [48].

**Figure 4:**
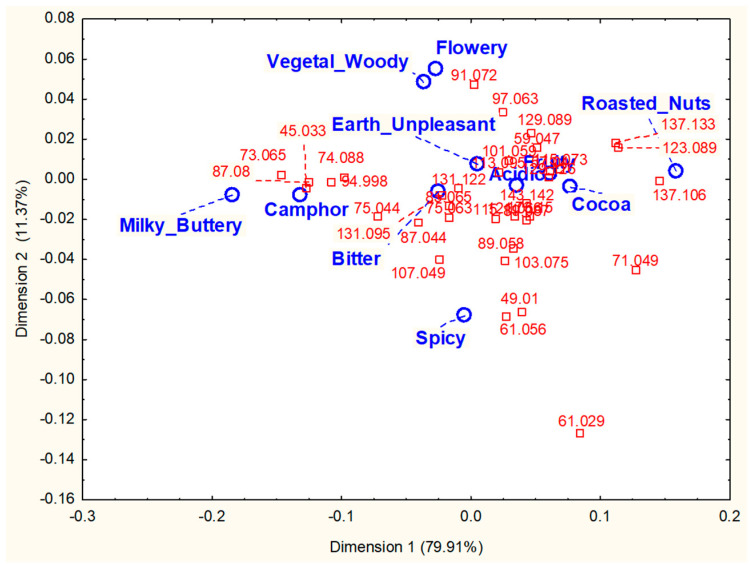
Correspondence analysis of the abundance while dominance (AWD) scores obtained by a panel of 12 assessors (3 replicates) for aroma release (PTR-ToF-MS, 35 ions) and aroma perception (TDS, 11 predefined descriptors) of a dark chocolate [134].

Nevertheless, the method was used to investigate the relationships between sensory attributes and released aromas in eight dark chocolates differing in characterized sensory properties (Table 1, [133,134,135]). In the nosespace analyses conducted with a PTR-ToF-MS, 35 ions tentatively identified to the molecular MH^+^ ions of aroma compounds were significantly detected. They were characteristic of chocolate aroma, notably with pyrazines, Strecker aldehydes, furanones, and terpenes. Their masses ranged from *m*/*z* 45.033 (acetaldehyde) to *m*/*z* 173.150 (ethyl octanoate). Meanwhile, using TDS, eleven predefined sensory attributes were evaluated, among which nine were aroma descriptors (e.g., “fruity”, “milky-buttery”, “roasted nuts”, etc.). Despite huge inter-individual variability in terms of release behaviors and dominance perceptions, the averaged release curves displayed some temporal effects, with significant longer Tmax for some molecules. These could be hardly related to the successive panel dominance rates that reached significance. However, the CA of the AWD scores’ contingency tables for each chocolate revealed some correspondences between attributes and ions. Thus, for one chocolate, the “milky-buttery” note was clearly associated with butan-2-one (*m*/*z* 73.065), and the “roasted nuts” attribute was associated with di- and tri-substituted pyrazines (*m*/*z* 123.089 and *m*/*z* 137.106, respectively; Figure 4, [133,134,135]).

However, the main information inferred from the CA maps was that dominant sensations were essentially due to a particular mixture of odorants released at a certain time typical of each product. In fact, the combinatorial code of aromas that could explain most of the dominant sensations depended on the product. The proportion of the different odorants in the mixtures perceived by a panelist over time during consumption appeared more important than specific odorants that could exceptionally explain specific sensations [134,135]. In another study on three dark chocolates differing in sensory properties, sensory evaluation was conducted with both TDS and TCATA procedures, while the nosespace of 16 assessors was concurrently measured in duplicates using PTR-ToF-MS (Table 1, [53]). The release of 19 discriminant aroma compounds whose MH^+^ masses ranged from *m*/*z* 49.011 (methanethiol) to *m*/*z* 143.145 (nonanal) was followed, and six predefined sensory attributes (“fruity”, “dry fruit”, “roasted”, “woody”, “cocoa”, and “spicy”) were evaluated as dominant sensations (TDS) or citable attributes (TCATA). The three chocolate samples were clearly distinguished at the nosespace and sensory levels, whatever the temporal sensory method used [53]. The CA of AWD indices calculated for each sensory method made it possible to draw the same conclusions as previously stated. The links between ions and dominant sensations (TDS) or cited attributes (TCATA) displayed various combinatorial codes of aromas that could explain the different perceptions, allowing samples differentiation [53]. However, a multiblock analysis of the paired aroma-release and sensory temporal data revealed that the chocolates discrimination obtained with TDS was more similar to the discrimination displayed in nosespace than in the TCATA [136]. In this case, TDS appeared to better reflect the nosespace data [136].

Two recent studies using TI as the sensory methodology afforded interesting results. Thus, discontinuous TI evaluation of the mint aroma and sweetness of chewing gums was conducted, with concurrent PTR-ToF-MS in-nose release monitoring of VOCs linked to mint aroma: monoterpenes (C_10_H_16_H^+^) at *m*/*z* 137.133, menthol (C_10_H_19_^+^, dehydrated) at *m*/*z* 139.148, menthofuran (C_10_H_14_OH^+^) at *m*/*z* 151.112, and menthone/1,8-cineole (C_10_H_18_OH^+^) at *m*/*z* 155.144 (Table 1, [137]). Significant differences for aroma release were found between Chinese and European panelists, this ethnicity effect being also significant for mint aroma and sweetness perception. Contrastingly, no gender effect was evidenced [137]. Moreover, it was found that measured physiological parameters (oral cavity volume, salivary flow, breath acetone concentration, and fungiform papillae density on the tongue) did not explain the relationships between aroma release in the nosespace and associated perception [137]. In a different register, condiments like spreads, mayonnaises, or vinaigrettes are generally consumed with solid carrier foods like bread or vegetables, making composite foods. In these composite foods, the interactions between condiment and carrier food are susceptible to affecting aroma release and perception during consumption. Lemon-flavored mayonnaises were evaluated in the absence or the presence of solid carrier foods (bread or potatoes) using TI for the lemon flavor and PTR-ToF-MS for the in-nose release of corresponding added aroma molecules, namely citral and limonene (Table 1, [138]). When mayonnaises were evaluated alone, a perfect correspondence between aroma release and perception was observed, modulated by the mayonnaise’s viscosity (Table 1, [138]). On addition of carriers (bread or potatoes), in-nose aroma (citral and limonene) release increased, while perceived lemon aroma intensity decreased [138]. The increasing aroma release in the nosespace could be explained by varying masticatory behavior and/or a surface area increase induced by the presence of the carrier food. The concomitant perception decrease clearly highlighted a cross-modal texture–aroma interaction [138]. Demonstration that not only physicochemical characteristics of foods but also cross-modal interactions play a role in flavor perception of composite foods was further confirmed in a study on chocolate-hazelnut spreads consumed with or without carrier (bread or wafer) foods (Table 1, [139]). If the fat and sugar content of the spreads had only a limited effect on in vivo aroma release and perception, in contrast, the addition of carriers strongly affected aroma release for all target molecules and perception of the predefined corresponding attributes measured using TCATA. The addition of carriers to spreads resulted in an increasing aroma release in the nosespace (duration and intensity) and a decreasing aroma perception [139]. Thus, carrier food addition modulates aroma perception of composite foods by cross-modal texture–aroma interactions.

Three additional recent investigations are worth citation. Addition of oenological tannins in wines is supposed to provide wine stability, protection from oxidation, and sensory persistence. However, their incidence in red wines is controversial. In a study pairing PTR-ToF-MS nosespace and TDS sensory evaluation of pinot noir red wine (Table 1, [140]), it was found that oxidation resulted in decreasing dominance and persistence of the “fruity” attribute, while the dominance of the “maderized” and “prune” descriptors increased. Concurrently, a decrease in the fruity ester ethyl decanoate and an increase in oxidative Strecker aldehydes were noticed in the nosespace [140]. Addition of ellagitannins but not proanthocyanidins protected the wine from oxidation, preserving perception of fruitiness and preventing the increase in “maderized” notes. Moreover, ellagitannins increased the aroma persistence in the non-oxidized wine (Table 1, [140]).

The impact of capsaicin (a chemesthesis agent responsible for spiciness) on aroma release and simultaneous perception was investigated using a flavored (nutty note) solution of 3-methylbutanal (Table 1, [141]). It was found that capsaicin had no significant impact on the in-nose aroma-release concentration, while the presence of capsaicin significantly enhanced aroma perception by 45% during a 60 s observation. The capsaicin-enhanced aroma perception found in this study revealed a perceptive cross-modal (chemesthesis–aroma) interaction, although a halo-dumping effect could not be completely ruled out [141]. Therefore, to consolidate the knowledge on capsaicin’s effect on aroma perception, further studies conducted with fully trained participants on more congruent flavors (such as savory) should be envisaged [141].

Finally, in the worldwide context of salt reduction in food products for a healthier diet, two promising strategies based on odor-induced saltiness enhancement and heterogeneous distribution of flavor compounds were tested in four-layer, cream-based, hot flans (Table 1, [41]). Salt and an aroma mixture were added homogeneously or heterogeneously to the four-layer snacks according to an experimental design, yielding seven variants. Globally, the sensory results on aroma perception suggested that a homogeneous distribution of salt induced higher aroma intensity perception regardless of the added aroma distribution. Concurrently, the measured in-nose aroma levels revealed a higher release for the products with a homogeneous salt distribution compared to the products with a heterogeneous one [41]. Regardless of the added aroma distribution, products with heterogeneous salt distribution were perceived as significantly saltier than products with the homogeneous one. The products containing salt in only the outer layer were perceived as the saltiest. This salty taste enhancement could be due to the initial strong dominance of the salty sensation at the very beginning of the eating process as measured using TDS [41]. Thus, a heterogeneous distribution of salt could constitute an interesting strategy to enhance saltiness in reduced-salt food. Moreover, aroma release was not affected by aroma compound distribution but only by salt distribution in the food product, probably revealing a salting-out effect. The involved mechanisms based on a combination of physicochemical and perceptual effects need further investigations to be fully understood [41].

**Table 1:** Dynamic nosespace and sensory analyses conducted simultaneously.

Food Product	Type of Variation Studied	Number of Panelists *(replicates)*	Sensory Method	Instrumental Method	Relevant Findings	Reference
Carvone-flavored gelatin gels	Carvone concentration	14	TI	APCI	Linear relationship between stimulus and perception. Effect of speed of eating on adaptation to the stimulus.	Hollowood et al., 2000 [115]
Rosemary-flavored solid food	Flavoring time in process	6	TI	APCI	Correlation between aroma release and simultaneous perception of rosemary flavor.	Cook et al., 2005 [5]
Flavored yogurt	Fat content	10 *(5)*	Modified DTI *(questionnaire)*	APCI	Quicker and greater aroma release in low-fat yogurts but with less persistence. Lipophilic compounds more affected by fat for Imax but not for Tmax or persistence. Significant sensory differences (intensity and timing) evidenced. Differences in particle size and viscosity might also affect aroma release.	Brauss et al., 1999 [118]
Flavored milk	Fat content	98 *(4)*	Paired test *	APCI	Good correlation between aroma delivery and perception, with higher intensity in low-fat milk.	Shojaei et al., 2006 [119]
Flavored liquid emulsions	Fat content	6 *(3)*	TI	PTR	Significant effect of fat on release and perception and on pre- and post-swallow events.	Frank et al., 2011 [120]
Flavored model gels	Gelatin concentration	11	TI	APCI	Decreased aroma perception on increasing gelatin concentration with no significant differences in aroma release (texture–aroma cross-modal interaction). Correlation of sensory data with the different rates of aroma release in the different gels.	Baek et al., 1999 [123]
Flavored whey protein gels	Whey protein content	10 *(3)*	TI	APCI	Texture of gels determines perception of aroma intensity rather than in-nose aroma concentration. Texture–aroma cross-modal interactions evidenced.	Weel et al., 2002 [124]
Flavored model gel	Whey protein content	7 *(3)*	TI	PTR	Correlation between individual-specific consumption patterns and respective sensory perception. Correlation between gel texture and release patterns and corresponding aroma perception.	Mestres et al., 2006 [17]
Flavored, stirred yogurts	Viscosity (protein content and mechanical treatment)	8 *(4)*	3-points DTI **	APCI	Complex viscosity of yogurts influenced in-nose release and perception. Aroma release and perception stronger in low-viscosity yogurts than in high-viscosity ones. Aroma release and perception more influenced by mechanical treatment than by protein composition.	Saint-Eve et al., 2006 [126]
Flavored candies	Gelatin concentration; melting or chewing protocols	12 *(4)*	TDS	PTR	Highest aroma release (Imax) obtained with low gelatin content. Aroma release determined by interaction between product properties and oral behavior. Relationships between dynamics of release and perception established for temporal parameters.	Déléris et al., 2011 [125]
Flavored model gels	Thickener type and level; sugar and flavor level	6	TI	APCI	Significant correlation between stimulus and perception depending on gel strength. Effect of speed of eating on adaptation to the stimulus.	Linforth et al., 1999 [117]
Flavored model custards	Thickener and sugar level	7 *(6)*	TI	APCI	Perceptual sweetness–aroma interactions, whatever the texture of the desserts.	Lethuaut et al., 2004 [127]
Mint-flavored commercial chewing gum	Gum type	11 *(3)*	TI	APCI	Decreasing perception of mint flavor followed sucrose release rather than menthone release. Sweetness–aroma cross-modal interaction evidenced.	Davidson et al., 1999 [128]
Mint-flavored carbonated beverages	CO_2_ and sugar level	4 *(8)*	3-points DTI **	PTR	CO_2_ increased aroma release and perception regardless of sugar content. Perceptual sweetness–aroma interactions evidenced. Impact of sugar content on aroma release but not on perception for carbonated beverages.	Saint-Eve et al., 2009 [129]
Soft cheeses	Cheese type	15 *(3)*	TI	APCI	Correlation between temporal sulfury notes and main sulfur compounds’ temporal release. Sensitivity and technical limitations of APCI evidenced for highlighting other relationships.	Salles et al., 2003 [130]
Alcoholic beverages	Spitting out or Swallowing	10 *(4)*	TDS	PTR	On swallowing, aroma-release data partly accounted for the observed differences in perception.	Déléris et al., 2011 [131]
Espresso coffee	Roasting degree and sugar level	18 *(3)*	TDS	PTR-ToF	Significant effect of roasting on release and perception. Sweet taste–smell perceptual interaction.	Charles et al., 2015 [132]
Commercial flavored (“garlic and herbs”) fresh cheeses	Brand	16 *(2)*	TDS	PTR-ToF	Significant relationships between dominant sensations and released aromas highlighted in correspondence analyses (CA) of abundance while dominant (AWD) indices ***.	Schlich et al., 2015 [48]
Dark chocolate	Categorized sensory properties	12 *(3)*	TDS	PTR-ToF	Sensory categories confirmed by TDS. Significant relationships between dominant sensations and released aromas revealed by CA of AWD indices ***.	Deuscher et al., 2019 [133,134,135]
Dark chocolate	Sensory properties	16 *(2)*	TDS and TCATA	PTR-ToF	Samples differentiation confirmed by sensory (both TDS and TCATA) and aroma release. Relationships between dominant sensations (TDS) or cited attributes (TCATA) and released aromas revealed by CA of AWD indices ***.	Le Quéré et al., 2021 [53]
Commercial mint chewing-gums	Ethnicity, gender, and physiology	29 *(3)*	DTI	PTR-ToF	Effect of ethnicity on correlated aroma release and perception not explained by physiological parameters. No gender effect.	Pedrotti et al., 2019 [137]
Composite food (lemon-flavored mayonnaise on carrier foods)	Fat content and viscosity level; carrier food	14 *(3)*	TI	PTR-ToF	Increasing mayonnaise viscosity resulted in lower aroma release and perception. Addition of carriers increased in-nose aroma release while decreasing perceived aroma intensity. Carrier addition modulates aroma perception of composite foods by cross-modal texture–aroma interactions.	Van Eck et al., 2021 [138]
Composite food (flavored chocolate-hazelnut spreads on carrier foods)	Fat and sugar content; carrier food	8 *(3)*	TCATA	PTR-ToF	Carriers’ attributes perceived at beginning of consumption, while spreads’ attributes perceived after swallowing. Limited effect of fat and sugar content on aroma release and perception. Addition of carriers increased aroma release (duration and intensity) and decreased perception. Cross-modal texture–aroma interactions evidenced.	Gonzalez-Estanol et al., 2023 [139]
Red wine	Oenological tannins; wine oxidation	17 *(2)*	TDS	PTR-ToF	Addition of ellagitannin extract in wine impacts the dynamic of sensations of oxidized wine and the length of aroma release in mouth and preserves fruitiness under oxidative conditions.	Pittari et al., 2022 [140]
Flavored solutions	Capsaicin present or not	15 *(3)*	Sequential profiling	APCI	No significant impact of capsaicin on aroma release but aroma perception significantly higher. Capsaicin enhanced saliva flow.	Yang et al., 2021 [141]
Flavored, four-layer, hot flans	Odor-induced saltiness enhancement; heterogeneous distribution of flavor compounds	15 *(3)*	ATI ****	PTR-ToF	Increased aroma release and perception in products salted homogeneously. Increased saltiness in heterogeneously salted products regardless of aroma distribution.	Emorine et al., 2021 [41]

* paired comparison test: does a significant perceivable difference in flavor intensity exist between samples? Not a dynamic method but conducted simultaneously with the nosespace analyses of 98 individuals. ** 3-points DTI: discrete time intensity of overall olfactory intensity measured at three consumption times, i.e., intake, swallowing, and 60 s after intake (persistence). *** AWD: calculated index of abundance of each volatile compound while a given attribute is dominant [48]. See text for a complete description. **** ATI: alternate TI, equivalent to DTI evaluating only two attributes (salty taste and one specific aroma).

Dynamic instrumental and sensory analyses conducted simultaneously revealed several relationships between aroma release and aroma perception, and these relationships were modulated by food structure and texture, perceptual cross-modal interactions, and, last but not least, inter-individual variability in release and perception behaviors. These aspects are discussed below. However, these analyses conducted concurrently present the main drawback of being realized during individual sessions only due to instrumental constraints. Typically conducted with a dozen of panelists and with the necessary replicates, data acquisition on several food variants can be rather lengthy. Moreover, the sensory and instrumental data used for establishing the relationships are generally treated as averaged data over the whole panel, which partially prevents taking into account the true temporality of the phenomena and the inter-individual variability. This is a challenge for the future.

## 5. Aroma Release and Aroma Perception: Is the Link So Close?

As outlined above, relating in vivo aroma release and aroma perception is generally not straightforward. A simple relationship between aroma release and corresponding perception is rarely observed, essentially in very simple systems (e.g., aromatized solutions or gels) far from ecologically relevant foodstuffs, which are unfamiliar and likely unpleasant. As soon as food systems are made more complex to resemble real foods or when real foods are considered, interactions of aroma with the food ingredients within the food matrix occur, which results in variable consequences on aroma release and perception [7,142]. Moreover, a given sensory attribute may result from the combined action of several aroma compounds, and a particular ion detected using DIMS techniques may correspond to several distinct molecules. Furthermore, perceptual interactions between odorants add another sensory dimension, as confirmed in recent studies on wine [143,144]. Also linked to more complex systems, perceptive cross-modal interactions between aroma and other sensory modalities (texture, taste, chemesthesis, or trigeminal sensations, although rarely investigated for the latter) have been evidenced. Finally, all the effects on perception are complicated by the interactions with human oral physiology and oral food-processing behavior that result in huge inter-individual variability in aroma release and perception [3].

### 5.1. The Food Matrix

Food systems comprise various ingredients (carbohydrates, lipids, and proteins) that influence their structure and texture. Aroma compounds interact with these major food ingredients through different mechanisms such as phase partitioning and binding or altered diffusion, and these interactions may affect aroma compounds volatility and hence their availability in the retronasal pathway for perception [142]. Any formulation modification using, e.g., fat replacers, thickeners, or sweeteners, is thus susceptible to modifying aroma release and perception by changing the nature of the interactions involved [7].

Proteins interact with aroma compounds through reversible (mainly hydrophobic interactions and hydrogen bonds) or irreversible (covalent) binding. The reversible binding strength is dependent on the aroma compound’s hydrophobicity, hence inducing variable effects on release and perception [7] that are exemplified in several of the results presented above (Table 1). Furthermore, if aldehydes are known to irreversibly bind to proteins through covalent binding, a recent study has shown that aroma compounds of various chemical classes may covalently bond to proteins during thermal processing through Schiff base, Michael addition, and disulfide linkages [145].

The addition of carbohydrates clearly modifies the structure of the matrix, affording viscosity changes that modulate texture. Molecular interactions between polysaccharides and aroma compounds may also occur. However, these nonspecific molecular interactions have a moderate impact on aroma release compared to the effects caused by the changes in viscosity [7]. Moreover, the respective characteristics of both the carbohydrates and the aroma compounds (volatility, polarity, and hydrophobicity) induce various retention and release effects that do not allow drawing a clear picture of carbohydrate–aroma interactions. However, carbohydrate concentration plays a major role, and aroma perception was shown to decrease rapidly when this concentration was higher than a critical concentration, i.e., c*, which is the point at which the viscosity of the system abruptly increases. It corresponds to the transition from a solution where macromolecules can move freely to a more organized gel. The reduced diffusion of aroma compounds in harder gels produces a decreasing release in the mouth and thus a decrease in perception [146]. Generally, an increase in viscosity seems to result in a decrease in the intensity of aroma perception but not necessarily in a decreasing aroma release. Thus, in gelified systems such as yogurts, Saint-Eve et al. [126] showed that for the same protein concentration, a decrease in viscosity induced by the application of a mechanical treatment resulted in an increase in aroma release and the intensity of aroma perception (Table 1). In model dairy desserts textured with carrageenan variants and sweetened with sucrose, Lethuaut et al. [127] showed that changes in sweetness and texture induced changes in aroma perception, while aroma release remained largely unaffected, highlighting perceptual sweetness–aroma interactions (Table 1). A perceptual interaction between odor and oral texture was also evidenced when a cream odor was presented ortho- or retronasally to assessors while milk-like foods with different viscosities were simultaneously present in the mouth [147]. Thus, the perceived aroma intensity decreased with increasing viscosity of the liquid irrespective of whether or not the odor was presented ortho- or retronasally at a constant concentration. Remarkably, the odor stimulus also increased the perceived intensities of thickness and creaminess of the fluid in the mouth but only when presented in the retronasal mode, that is, as if the odor had originated from the liquid [147]. Sucrose itself affects aroma release, and both an increase and a decrease in release following the addition of sucrose to water were noticed. Increase in sucrose concentration caused an increased release of the more volatile compounds and a decreased release of the less volatile ones. However, no significant differences in sensory perception were evidenced [7]. An increase in the mole fractions of the highly volatile compounds in the liquid phase on addition of sucrose could explain the increased aroma release [7]. An increased release of more hydrophilic compounds can also be explained by stronger competition with sucrose for water molecules [146]. The decrease observed for the less-volatile compounds was attributed to an increase in viscosity, which may affect the diffusion of aroma molecules [7].

Lipids influence the flavor of foods through various effects on multimodal aspects of perception (mouthfeel, taste, and aroma). For aroma compounds, lipids influence their perception by direct impacts on food texture and aroma release [142]. Lipids induce phase partitioning, and aroma compounds are distributed between fat and the aqueous phase, following partition governed by hydrophobicity [7,142]. For most aroma compounds that are essentially lipophilic, the effect of fat on their release is greater than that of other ingredients. Thus, small changes in fat content have significant effects on the volatility of aroma compounds and thus their perception (Table 1). However, these effects are largely dependent on the compound’s hydrophobicity and polarity, with the vapor pressure of the more polar compounds decreasing only slightly [7]. Thus, for a mixture of aroma compounds exhibiting different polarities, the relative proportions of their release change significantly with a changing fat ratio, inducing changes in overall aroma perception [7]. Furthermore, to explain aroma perception in lipid-containing food, it is also necessary to consider the release rate of aroma compounds that significantly influences their odor thresholds; these are influences that, once more, are modulated by their hydrophobicity and polarity [7,142]. Moreover, the nature of fat affects aroma release [142,148], and the presence of emulsifying proteins at the interface of oil in water emulsions affects the hydrophobicity-dependent release of aroma compounds through the additional protein–aroma interactions described above [7,142]. Emulsion properties such as droplet size and distribution, microstructure, and viscosity also affect aroma release and perception [142].

Among other food ingredients, phenolic compounds may be involved in interactions with aroma compounds that may affect aroma release and perception. This effect is particularly relevant for wine and olive oil, where the polyphenols content is significant. However, the real effect of these macromolecules on aroma release and perception should be considered through the prism of their associations with proteins [7,142]. Salt (NaCl) is another ubiquitous food ingredient important for food palatability but also for technological issues (inhibition of pathogen microorganisms’ growth, texture improvement, and involvement in enzymatic and biochemical reactions during food maturation). The salting-out effect that results in increasing the release of volatile compounds is dependent on the chemical nature of the volatiles, which may result in modulations of aroma perception [7]. Moreover, in association with lipids, salt modifies in-mouth aroma release and perception [111,149]. Furthermore, health concerns lead to reducing sodium content in foodstuffs and to developing low-salt foods. One of the main consequences of decreasing salt content is the modification of the sensory characteristics of the product through a perceptive aroma–saltiness interaction [150].

For interested readers, detailed consequences of food ingredient interactions with aroma compounds on release and perception may be found in dedicated treatises (e.g., [151,152,153]) and specialized reviews (e.g., [7,142,146,154]).

### 5.2. Cross-Modal Interactions

Once halo-dumping effects are ruled out, for instance, with adequate training of the panelists, perceptive cross-modal interactions can be evidenced, as exemplified in several studies described above (Table 1). Several perceptive aroma–texture interactions were evidenced as a consequence of altering food viscosity by modifying the content or the nature of thickeners. Moreover, these interactions should always be considered for composite foods when the use of a carrier food systematically modifies the aroma perception of the spreads. Cross-modal aroma–taste interactions were also evidenced essentially with several examples of sweetness–aroma interaction that can be found in Table 1. For a long time, it has been demonstrated that an odor can enhance taste perception and, conversely, that a taste can increase an odor intensity [155]. It has been also established that such perceptual interactions occur when odor and taste are congruent [150]. Thus, sucrose but not salt and citric acid significantly increased the perceived intensity of the sweet-related aromas vanillin, citral, and furaneol [156]. Concomitantly, the salt-related sardine aroma induced an increased saltiness perception in low-salt solutions, while a sweet-related carrot aroma did not [157]. This phenomenon, known as odor-induced taste enhancement (OITE), is considered to be an efficient strategy to compensate for sugar reduction [158] and salt reduction [159] in food while maintaining acceptability for the consumers. It is noteworthy that these cross-modal interactions seem processed in high-level integratory areas of the brain with potential top-down effects on primary sensory regions [1].

### 5.3. Inter-Individual Variability

For a long time, huge inter-individual variability in aroma release and perception has been evidenced [3], which requires a significant number of panelists and replicates to infer relevant results, however, often only at panel level. The properties of the food matrix, which undergoes physicochemical interactions with aroma compounds, as described above, and oral physiological characteristics and their interactions during food oral processing are the main drivers that produce inter-individual variability. It is out of the scope of the present review to exhaustively detail all the implications of these complex relationships on aroma release and perception, and the interested reader is invited to refer to dedicated reviews (e.g., [3,12,23,160]) and treatises (e.g., [152,153]). Only the most salient causes of variation will be described briefly.

Obviously, the first cause of variation in odor perception comes from the human genome. Polymorphism in olfactory receptors genes results in different genotypes and phenotypes that alter odor perception in terms of both intensity and pleasantness [161]. Whether the existence of these phenotypes could explain specific anosmia and hyposmia or not is still debated [161]. Following food intake and before being transferred to the breath, aroma compounds are released in saliva. Saliva variations in both flow and volume contribute to varying aroma release in the oral cavity during food oral processing. Moreover, saliva composition in proteins and enzymes plays a major role. Thus, a low saliva flow and a low α-amylase content were found responsible for a higher aroma release [162]. Variations in the salivary proteome modulate sensory perception and in-mouth enzymes originating from the oral microbiote, saliva, or epithelial cells are able to metabolize aroma compounds in the time course of food consumption (e.g., [163,164,165]). Mouth coating of residual food that sticks to the oral surface after food ingestion, which is dependent on matrix composition, and selective adsorption of aroma compounds to the oral and pharyngeal mucosa modify aroma compounds immediate availability and participate in “after-smell” perception [3] or persistence (e.g., [160,164]). With solid food, salivation and mastication combine to form a food bolus ready to be swallowed. The aroma compounds transported from the saliva to the air phase in the mouth are transferred to the upper airways essentially on swallowing. This so-called “swallow-breath” comes with an aroma pulse [166] that generally presents the highest aroma-release signal, whatever the type of food [167]. Swallowing requires forcing the bolus or the liquid beverage into the pharynx by a tongue movement while the velum retracts and elevates, preventing food material from being swallowed the wrong way [12]. Nevertheless, as already outlined above [17], some individuals release aroma during the chewing phase, opening their velum and allowing important aroma transfer to the nose on each masticatory pulse before swallowing [146]. This source of variation in aroma release is even further complicated by the observation of groups of people releasing either a high amount (high-releaser group, HRG) or a very low quantity (low-releaser group, LRG) of aroma [168], whatever the food product, confirming previous observation [169]. HRG subjects were better discriminated from LRG ones by a higher chewing activity (number of chewing cycles, chewing duration, maximal amplitude, and total muscle work), confirming previous results (e.g., [15]); higher mouth coating; and higher velum-opening frequency [168]. A multiblock partial least-square regression conducted on the different physiological and behavioral oral processing datasets (masticatory behavior, bolus rheology, saliva composition and flux, mouth coating, and bolus moistening) confirmed the importance of masticatory behavior on aroma release [162]. Although breath flow was not measured, and despite some contradictory results [160], the influence of breathing flow on aroma release could also account for the difference between the two groups. Thus, during wine drinking, higher releasers were characterized by higher volume of air breathed out [170], indicating that breathing capacity could also be important in the aroma-release process [160], confirming earlier results (e.g., [67]).

Finally, as another source of variability, recent studies highlight the importance of perireceptor molecular events in the modulation of aroma perception [165]. Thus, metabolism of odorant molecules by odorant metabolizing enzymes was found to be effective in the human nasal cavity, affecting odorant perception [171], and in vivo odorant competitive metabolism was demonstrated to be involved in the human olfactory process, influencing the intensity and quality of aroma perception [172].

## 6. Concluding Remarks and Future Trends

Aroma perception is a complicated phenomenon that results from a dynamic interplay between aroma release from a food matrix, where interactions of aroma compounds with food ingredients modulate the release; human physiology and food oral processing behavior; and perceptive cross-modal interactions. However, the resulting aroma release does not always explain sensory perception due to other physiological mechanisms at the peripheral and central levels in the brain. Moreover, aroma perception contributes to a unitary flavor perception that arises from the central integration of multiple sensory inputs, among which aroma and taste are of prime importance. Thus, odor/taste integration that constructs the first level of flavor perception depends on neural processes that occur in chemosensory regions of the brain [173].

As aroma release and aroma perception are dynamic processes, the potential link between release and perception can only be understood if instrumental methods that apprehend the time dimension of aroma release are paired with dynamic sensory methods, preferably using a concurrent protocol. Mass spectrometry methods based on DIMS techniques are satisfactory, but only the most sensitive instruments allow untargeted analyses of real foods. Currently developed on the PTR-ToF-MS technology, a gain in sensitivity is desirable to be able to follow key odorants that are often found in trace amount and are simply not detected in vivo using the current instruments. SESI-MS has to prove its usefulness in the near future, while the less-expensive ion mobility spectrometry (IMS) technique has recently demonstrated some potentialities [174]. Available temporal sensory methods should also be ameliorated, particularly as they better take into account the temporality of the phenomena and inter-individual variability. A method such as the temporal order of sensations that apprehends the most salient perception moments could be an interesting alternative to the other temporal sensory methods used so far. Efforts on combined sensory/instrumental data treatments should also be undertaken beyond the current models based on the AWD index [48] or multiblock analyses [136].

Finally, the valuable results reviewed above have elucidated several relationships between aroma release and aroma perception, often highlighting perceptive cross-modal interactions. However, a perfect understanding of all the relationships that link food’s oral processing, aroma release, and aroma perception is still incomplete because of the complexity of the phenomena. Particularly, as inter-individual physiological variability leads to differences in aroma release, which affects aroma perception, future research should take into account these variations. Moreover, most of the results were obtained using a single-bite/sip food intake of model or simple foodstuffs. Nevertheless, heterogeneous foods or composite foods modify oral processing behavior, sensory perception, and food intake [175], and recent results obtained for aroma release and perception of composite foods have highlighted aroma–texture-perceptive interactions [138,139]. Therefore, it should be interesting to consider food’s oral processing and related aroma release and perception during more natural food consumption behavior, investigating multi-bites/sips food intake conditions or even a whole meal. Available dynamic instrumental and sensory methods have the potentiality to tackle this new paradigm; with associated chemometrics development, a new chapter of flavoromics that connects the chemical profiling of flavor-impact compounds to the sensory science should be written.

## Data Availability

Not applicable.

## References

[1] Sinding C., Thibault H., Hummel T., Thomas-Danguin T. (2021). Odor-Induced Saltiness Enhancement: Insights Into The Brain Chronometry Of Flavor Perception. Neuroscience.

[2] Schieberle P., Hofmann T., Jelen H. (2011). Mapping the combinatorial code of food flavors by means of molecular sensory science approach. Food Flavors: Chemical, Sensory and Technological Properties.

[3] Buettner A., Beauchamp J. (2010). Chemical input—Sensory output: Diverse modes of physiology–flavour interaction. Food Qual. Pref..

[4] Rochelle M.M., Prévost G.J., Acree T.E. (2018). Computing Odor Images. J. Agric. Food Chem..

[5] Cook D.J., Hollowood T.A., Linforth R.S.T., Taylor A.J. (2005). Correlating instrumental measurements of texture and flavour release with human perception. Int. J. Food Sci. Technol..

[6] Dunkel A., Steinhaus M., Kotthoff M., Nowak B., Krautwurst D., Schieberle P., Hofmann T. (2014). Nature’s Chemical Signatures in Human Olfaction: A Foodborne Perspective for Future Biotechnology. Angew. Chem. Int. Edit..

[7] Guichard E. (2002). Interactions between flavor compounds and food ingredients and their influence on flavor perception. Food Rev. Int..

[8] Monteleone E., Dinnella C., Hort J., Kemp S.E., Hollowood T. (2017). General considerations. Time-Dependent Measures of Perception in Sensory Evaluation.

[9] DeRovira D. (1996). The dynamic flavor profile method. Food Technol..

[10] Piggott J.R. (2000). Dynamism in flavour science and sensory methodology. Food Res. Int..

[11] Seuvre A.-M., Voilley A., Buettner A. (2017). Physico-Chemical Interactions in the Flavor-Release Process. Springer Handbook of Odor.

[12] Salles C., Chagnon M.-C., Feron G., Guichard E., Laboure H., Morzel M., Semon E., Tarrega A., Yven C. (2011). In-Mouth Mechanisms Leading to Flavor Release and Perception. Crit. Rev. Food Sci. Nutr..

[13] Salles C., Benjamin O., Buettner A. (2017). Models of the Oral Cavity for the Investigation of Olfaction. Springer Handbook of Odor.

[14] Tarrega A., Yven C., Sémon E., Salles C. (2011). In-mouth aroma compound release during cheese consumption: Relationship with food bolus formation. Int. Dairy J..

[15] Tarrega A., Yven C., Sémon E., Salles C. (2008). Aroma release and chewing activity during eating different model cheeses. Int. Dairy J..

[16] Buettner A., Otto S., Beer A., Mestres M., Schieberle P., Hummel T. (2008). Dynamics of retronasal aroma perception during consumption: Cross-linking on-line breath analysis with medico-analytical tools to elucidate a complex process. Food Chem..

[17] Mestres M., Kieffer R., Buettner A. (2006). Release and Perception of Ethyl Butanoate during and after Consumption of Whey Protein Gels: Relation between Textural and Physiological Parameters. J. Agric. Food Chem..

[18] Roberts D.D., Pollien P., Yeretzian C., Lindinger C., Deibler K.D., Delwiche J. (2004). Nosespace analysis with proton-transfer-reaction mass spectrometry: Intra- and interpersonal variability. Handbook of Flavor Characterization.

[19] Biasioli F., Yeretzian C., Märk T.D., Dewulf J., Van Langenhove H. (2011). Direct-injection mass spectrometry adds the time dimension to (B)VOC analysis. Trac-Trends Anal. Chem..

[20] Taylor A.J., Beauchamp J.D., Langford V.S., Beauchamp J.D. (2021). Non-destructive and High-Throughput—APCI-MS, PTR-MS and SIFT-MS as Methods of Choice for Exploring Flavor Release. Dynamic Flavor: Capturing Aroma Using Real-Time Mass Spectrometry.

[21] Taylor A.J., Linforth R.S.T. (1996). Flavour release in the mouth. Trends Food Sci. Technol..

[22] Taylor A.J., Linforth R.S.T., Harvey B.A., Blake A. (2000). Atmospheric pressure chemical ionisation mass spectrometry for in vivo analysis of volatile flavour release. Food Chem..

[23] Taylor A.J. (2002). Release and transport of flavors in vivo: Physicochemical, physiological, and perceptual considerations. Comp. Rev. Food Sci. Food Saf..

[24] Beauchamp J., Zardin E., Buettner A. (2017). Odorant Detection by On-line Chemical Ionization Mass Spectrometry. Handbook of Odor.

[25] Hort J., Kemp S.E., Hollowood T. (2017). Time-Dependent Measures of Perception in Sensory Evaluation.

[26] Dijksterhuis G.B., Piggott J.R. (2000). Dynamic methods of sensory analysis. Trends Food Sci. Technol..

[27] Larson-Powers N., Pangborn R.M. (1978). Paired comparison and Time-Intensity measurements of the sensory properties of beverages and gelatins containing sucrose or synthetic sweeteners. J. Food Sci..

[28] Duizer L.M., Bloom K., Findlay C.J. (1997). Dual-attribute time-intensity sensory evaluation: A new method for temporal measurement of sensory perceptions. Food Qual. Pref..

[29] Pionnier E., Nicklaus S., Chabanet C., Mioche L., Taylor A.J., Le Quéré J.L., Salles C. (2004). Flavor perception of a model cheese: Relationships with oral and physico-chemical parameters. Food Qual. Pref..

[30] Kuesten C., Bi J., Feng Y. (2013). Exploring taffy product consumption experiences using a multi-attribute time–intensity (MATI) method. Food Qual. Pref..

[31] Pineau N., Schlich P., Cordelle S., Mathonniere C., Issanchou S., Imbert A., Rogeaux M., Etievant P., Koster E.P. (2009). Temporal Dominance of Sensations: Construction of the TDS curves and comparison with time-intensity. Food Qual. Pref..

[32] Castura J.C., Antúnez L., Giménez A., Ares G. (2016). Temporal Check-All-That-Apply (TCATA): A novel dynamic method for characterizing products. Food Qual. Pref..

[33] Visalli M., Galmarini M.V. (2022). Multi-attribute temporal descriptive methods in sensory analysis applied in food science: Protocol for a scoping review. PLoS ONE.

[34] Cliff M., Heymann H. (1993). Development and use of time-intensity methodology for sensory evaluation: A review. Food Res. Int..

[35] Meiselman H.L., Jaeger S.R., Carr B.T., Churchill A. (2022). Approaching 100 years of sensory and consumer science: Developments and ongoing issues. Food Qual. Pref..

[36] Chaya C., Hort J., Kemp S.E., Hollowood T. (2017). Continuous time-intensity. Time-Dependent Measures of Perception in Sensory Evaluation.

[37] Clark C.C., Lawless H.T. (1994). Limiting response alternatives in time-intensity scaling: An examination of the halo-dumping effect. Chem. Senses.

[38] Findlay C.J., Hort J., Kemp S.E., Hollowood T. (2017). Dual-attribute time-intensity. Time-Dependent Measures of Perception in Sensory Evaluation.

[39] Kuesten C., Hort J., Kemp S.E., Hollowood T. (2017). Time-intensity using discrete time points. Time-Dependent Measures of Perception in Sensory Evaluation.

[40] Hort J., Hollowood T., Kemp S.E., Hort J., Kemp S.E., Hollowood T. (2017). Summary. Time-Dependent Measures of Perception in Sensory Evaluation.

[41] Emorine M., Septier C., Martin C., Cordelle S., Sémon E., Thomas-Danguin T., Salles C. (2021). Salt and Aroma Compound Distributions Influence Flavour Release and Temporal Perception While Eating Hot-Served Flans. Molecules.

[42] Gordin H.H. (1987). Intensity variation descriptive methodology: Development and application of a new sensory evaluation technique. J. Sens. Stud..

[43] Jack F.R., Piggott J.R., Paterson A. (1994). Analysis of Textural Changes in Hard Cheese during Mastication by Progressive Profiling. J. Food Sci..

[44] Seo H.-S., Lee M., Jung Y.-J., Hwang I. (2009). A novel method of descriptive analysis on hot brewed coffee: Time scanning descriptive analysis. Eur. Food Res. Technol..

[45] Methven L., Rahelu K., Economou N., Kinneavy L., Ladbrooke-Davis L., Kennedy O.B., Mottram D.S., Gosney M.A. (2010). The effect of consumption volume on profile and liking of oral nutritional supplements of varied sweetness: Sequential profiling and boredom tests. Food Qual. Pref..

[46] Schlich P., Pineau N., Hort J., Kemp S.E., Hollowood T. (2017). Temporal Dominance of Sensations. Time-Dependent Measures of Perception in Sensory Evaluation.

[47] Schlich P. (2017). Temporal Dominance of Sensations (TDS): A new deal for temporal sensory analysis. Curr. Opin. Food Sci..

[48] Schlich P., Thomas A., Visalli M., Labarre D., Sémon E., Le Quéré J.-L., Taylor A.J., Mottram D.S. (2015). Collecting and analysing in vivo aroma release and perception by pairing nosespace PTR-ToF-MS and Temporal Dominance of Sensations. Flavour Science: Proceedings of the XIV Weurman Flavour Research Symposium.

[49] Varela P., Antúnez L., Carlehög M., Alcaire F., Castura J.C., Berget I., Giménez A., Næs T., Ares G. (2018). What is dominance? An exploration of the concept in TDS tests with trained assessors and consumers. Food Qual. Pref..

[50] Adams J., Williams A., Lancaster B., Foley M. Advantages and uses of check-all-that-apply response compared to traditional scaling of attributes for salty snacks. Proceedings of the 7th Pangborn Sensory Science Symposium.

[51] Jaeger S.R., Beresford M.K., Hunter D.C., Alcaire F., Castura J.C., Ares G. (2017). Does a familiarization step influence results from a TCATA task?. Food Qual. Pref..

[52] Meyners M., Castura J.C. (2018). The analysis of temporal check-all-that-apply (TCATA) data. Food Qual. Pref..

[53] Le Quéré J.L., Hélard C., Labouré H., Andriot I., Cordelle S., Schlich P. (2021). Nosespace PTR-MS analysis with simutaneous TDS or TCATA sensory evaluation: Release and perception of the aroma of dark chocolates differing in sensory properties. Proceedings of the American Chemical Society Annual Meeting.

[54] Ares G., Jaeger S.R., Antunez L., Vidal L., Gimenez A., Coste B., Picallo A., Castura J.C. (2015). Comparison of TCATA and TDS for dynamic sensory characterization of food products. Food Res. Int..

[55] Ares G., Alcaire F., Antúnez L., Vidal L., Giménez A., Castura J.C. (2017). Identification of drivers of (dis)liking based on dynamic sensory profiles: Comparison of Temporal Dominance of Sensations and Temporal Check-all-that-apply. Food Res. Int..

[56] Agudelo A., Varela P., Fiszman S. (2015). Methods for a deeper understanding of the sensory perception of fruit fillings. Food Hydrocoll..

[57] Bemfeito R.M., Rodrigues J.F., Silva J.G.e., Abreu L.R. (2016). Temporal dominance of sensations sensory profile and drivers of liking of artisanal Minas cheese produced in the region of Serra da Canastra, Brazil. J. Dairy Sci..

[58] Nguyen Q.C., Næs T., Varela P. (2018). When the choice of the temporal method does make a difference: TCATA, TDS and TDS by modality for characterizing semi-solid foods. Food Qual. Pref..

[59] Gonzalez-Estanol K., Cliceri D., Biasioli F., Stieger M. (2022). Differences in dynamic sensory perception between reformulated hazelnut chocolate spreads decrease when spreads are consumed with breads and wafers. Food Qual. Pref..

[60] Pecore S., Rathjen-Nowak C., Tamminen T. Temporal order of sensations. Proceedings of the 9th Pangborn Sensory Science Symposium.

[61] Vandeputte A., Romans J., Pineau N., Lenfant F. Innovative methods to assess the evolution of the sensory characteristics during the tasting of a full product portion (several bites). Proceedings of the 9th Pangborn Sensory Science Symposium.

[62] Visalli M., Mahieu B., Thomas A., Schlich P. (2020). Concurrent vs. retrospective temporal data collection: Attack-evolution-finish as a simplification of Temporal Dominance of Sensations?. Food Qual. Pref..

[63] Mahieu B., Visalli M., Thomas A., Schlich P. (2020). Using Free-Comment with consumers to obtain temporal sensory descriptions of products. Food Qual. Pref..

[64] Visalli M., Wakihira T., Schlich P. (2022). Concurrent vs. immediate retrospective temporal sensory data collection: A case study on lemon-flavoured carbonated alcoholic drinks. Food Qual. Pref..

[65] Linforth R.S.T., Taylor A.J. (1993). Measurement of Volatile Release in the Mouth. Food Chem..

[66] Taylor A.J., Linforth R.S.T., Maarse H., Van der Heij D.G. (1994). Methodology for measuring volatile profiles in the mouth and nose during eating. Trends in Flavour Research.

[67] Pionnier E., Chabanet C., Mioche L., Le Quéré J.L., Salles C. (2004). In Vivo Aroma Release during Eating of a Model Cheese: Relationships with Oral Parameters. J. Agric. Food Chem..

[68] Pionnier E., Semon E., Chabanet C., Salles C. (2005). Evaluation of the solid phase microextraction (SPME) technique for the analysis of human breath during eating. Sci. Aliment..

[69] Linforth R.S.T., Ingham K.E., Taylor A.J., Taylor A.J., Mottram D.S. (1996). Time course profiling of volatile release from foods during the eating process. Flavour Science: Recent Developments.

[70] Roberts D.D., Taylor A.J. (2000). Flavor Release.

[71] Berchtold C., Bosilkovska M., Daali Y., Walder B., Zenobi R. (2014). Real-time monitoring of exhaled drugs by mass spectrometry. Mass Spectrom. Rev..

[72] Gaugg M.T., Gomez D.G., Barrios-Collado C., Vidal-de-Miguel G., Kohler M., Zenobi R., Martinez-Lozano Sinues P. (2016). Expanding metabolite coverage of real-time breath analysis by coupling a universal secondary electrospray ionization source and high resolution mass spectrometry—A pilot study on tobacco smokers. J. Breath Res..

[73] Weber R., Kaeslin J., Moeller S., Perkins N., Micic S., Moeller A. (2023). Effects of a Volatile Organic Compound Filter on Breath Profiles Measured by Secondary Electrospray High-Resolution Mass Spectrometry. Molecules.

[74] Bean H.D., Mellors T.R., Zhu J., Hill J.E. (2015). Profiling Aged Artisanal Cheddar Cheese Using Secondary Electrospray Ionization Mass Spectrometry. J. Agric. Food Chem..

[75] Le Quéré J.L., Gierczynski I., Sémon E. (2014). An atmospheric pressure chemical ionization—Ion-trap mass spectrometer for the on-line analysis of volatile compounds in foods: A tool for linking aroma release to aroma perception. J. Mass Spectrom..

[76] Beauchamp J.D. (2021). Dynamic Flavor: Capturing Aroma Using Real-Time Mass Spectrometry.

[77] Le Quéré J.L., Lucchi G., Nollet L.M.L., Winkler R. (2022). Flavour and mass spectrometry. Mass Spectrometry in Food Analysis.

[78] Beauchamp J., Herbig J., Guthrie B., Beauchamp J., Buettner A., Lavine B.K. (2015). Proton-Transfer-Reaction Time-of-Flight Mass Spectrometry (PTR-TOFMS) for Aroma Compound Detection in Real-Time: Technology, Developments, and Applications. The Chemical Sensory Informatics of Food: Measurement, Analysis, Integration.

[79] Taylor A.J., Linforth R.S.T., Taylor A.J., Linforth R.S.T. (2010). On-line monitoring of flavour processes. Food Flavour Technology.

[80] Linforth R.S.T., Taylor A.J., Brown W.E., Bakker J. (1998). Volatile release from food in the mouth during the eating process. Book Volatile Release from food in the Mouth during the Eating Process, Proceedings of COST Action 96 “Interaction of food Matrix with Small Ligands Influencing Flavour and Texture”, Valencia, Spain, 14–16 November 1996.

[81] Linforth R.S.T., Taylor A.J. (1998). Apparatus and Method for the Analysis of Trace Constituents in Gases. Patent.

[82] Taylor A.J., Beauchamp J.D. (2021). Flavor Applications of Direct APCI-MS. Dynamic Flavor: Capturing Aroma Using Real-Time Mass Spectrometry.

[83] Sémon E., Gierczynski I., Langlois D., Le Quéré J.L., Ashcroft A.E., Brenton G., Monaghan J.J. (2003). Analysis of aroma compounds by atmospheric pressure chemical ionisation—Ion trap mass spectrometry. Construction and validation of an interface for in vivo analysis of human breath volatile content. Proceedings of the 16th International Mass Spectrometry Conference, Edinburgh, Scotland, 31 August–5 September 2003.

[84] Jublot L., Linforth R.S.T., Taylor A.J. (2005). Direct atmospheric pressure chemical ionisation ion trap mass spectrometry for aroma analysis: Speed, sensitivity and resolution of isobaric compounds. Int. J. Mass Spectrom..

[85] Zehentbauer G., Krick T., Reineccius G.A. (2000). Use of humidified air in optimizing APCI-MS response in breath analysis. J. Agric. Food Chem..

[86] Haahr A.M., Madsen H., Smedsgaard J., Bredie W.L.P., Stahnke L.H., Refsgaard H.H.F. (2003). Flavor Release Measurement by Atmospheric Pressure Chemical Ionization Ion Trap Mass Spectrometry, Construction of Interface and Mathematical Modeling of Release Profiles. Anal. Chem..

[87] Hatakeyama J., Taylor A.J. (2019). Optimization of atmospheric pressure chemical ionization triple quadropole mass spectrometry (MS Nose 2) for the rapid measurement of aroma release in vivo. Flavour Fragr. J..

[88] Kornbausch N., Debong M.W., Buettner A., Heydel J.-M., Loos H. (2022). Odorant Metabolism in Humans. Angew. Chem.-Int. Edit..

[89] Linforth R., Martin F., Carey M., Davidson J., Taylor A.J. (2002). Retronasal Transport of Aroma Compounds. J. Agric. Food Chem..

[90] Lindinger W., Hirber J., Paretzke H. (1993). An ion/molecule-reaction mass spectrometer used for on-line trace gas analysis. Int. J. Mass Spectrom. Ion Proc..

[91] Ellis A.M., Mayhew C.A. (2014). Proton Transfer Reaction Mass Spectrometry. Principles and Applications.

[92] Blake R.S., Monks P.S., Ellis A.M. (2009). Proton-Transfer Reaction Mass Spectrometry. Chem. Rev..

[93] Lindinger W., Fall R., Karl T.G. (2001). Environmental, food and medical applications of Proton-Transfer-Reaction mass spectrometry (PTR-MS). Adv. Gas-Phase Ion Chem..

[94] Lindinger W., Hansel A., Jordan A. (1998). On-line monitoring of volatile organic compounds at pptv levels by means of proton-transfer-reaction mass spectrometry (PTR-MS) Medical applications, food control and environmental research. Int. J. Mass Spectrom. Ion Proc..

[95] Yeretzian C., Jordan A., Brevard H., Lindinger W., Roberts D.D., Taylor A.J. (2000). Time-resolved headspace analysis by proton-transfer-reaction mass-spectrometry. Flavor release.

[96] Smith D., Španěl P., Demarais N., Langford V.S., McEwan M.J. (2023). Recent developments and applications of selected ion flow tube mass spectrometry (SIFT-MS). Mass Spectrom. Rev..

[97] Smith D., Španěl P. (2016). Status of selected ion flow tube MS: Accomplishments and challenges in breath analysis and other areas. Bioanalysis.

[98] Smith D., Španěl P. (2015). Pitfalls in the analysis of volatile breath biomarkers: Suggested solutions and SIFT–MS quantification of single metabolites. J. Breath Res..

[99] Xu Y., Barringer S. (2010). Comparison of Volatile Release in Tomatillo and Different Varieties of Tomato during Chewing. J. Food Sci..

[100] Ozcan G., Barringer S. (2011). Effect of Enzymes on Strawberry Volatiles during Storage, at Different Ripeness Level, in Different Cultivars, and during Eating. J. Food Sci..

[101] Castada H.Z., Barringer S.A. (2019). Online, real-time, and direct use of SIFT-MS to measure garlic breath deodorization: A review. Flavour Fragr. J..

[102] Langford V.S., Padayachee D., McEwan M.J., Barringer S.A. (2019). Comprehensive odorant analysis for on-line applications using selected ion flow tube mass spectrometry (SIFT-MS). Flavour Fragr. J..

[103] Mirondo R., Barringer S. (2016). Deodorization of Garlic Breath by Foods, and the Role of Polyphenol Oxidase and Phenolic Compounds. J. Food Sci..

[104] Blake R.S., Whyte C., Hughes C.O., Ellis A.M., Monks P.S. (2004). Demonstration of Proton-Transfer Reaction Time-of-Flight Mass Spectrometry for Real-Time Analysis of Trace Volatile Organic Compounds. Anal. Chem..

[105] Jordan A., Haidacher S., Hanel G., Hartungen E., Märk L., Seehauser H., Schottkowsky R., Sulzer P., Märk T.D. (2009). A high resolution and high sensitivity proton-transfer-reaction time-of-flight mass spectrometer (PTR-TOF-MS). Int. J. Mass Spectrom..

[106] Romano A., Cappellin L., Ting V., Aprea E., Navarini L., Gasperi F., Biasioli F. (2014). Nosespace analysis by PTR-ToF-MS for the characterization of food and tasters: The case study of coffee. Int. J. Mass Spectrom..

[107] Beauchamp J.D., Beauchamp J.D. (2021). Pushing the Boundaries of Dynamic Flavor Analysis with PTR-MS. Dynamic Flavor: Capturing Aroma Using Real-Time Mass Spectrometry.

[108] Jordan A., Haidacher S., Hanel G., Hartungen E., Herbig J., Märk L., Schottkowsky R., Seehauser H., Sulzer P., Märk T.D. (2009). An online ultra-high sensitivity Proton-transfer-reaction mass-spectrometer combined with switchable reagent ion capability (PTR+SRI−MS). Int. J. Mass Spectrom..

[109] Müller M., Piel F., Gutmann R., Sulzer P., Hartungen E., Wisthaler A. (2020). A novel method for producing NH4+ reagent ions in the hollow cathode glow discharge ion source of PTR-MS instruments. Int. J. Mass Spectrom..

[110] Swift S.J., Smith D., Dryahina K., Gnioua M.O., Španěl P. (2022). Kinetics of reactions of NH4+ with some biogenic organic molecules and monoterpenes in helium and nitrogen carrier gases: A potential reagent ion for selected ion flow tube mass spectrometry. Rapid Commun. Mass Spectrom..

[111] Boisard L., Tournier C., Sémon E., Noirot E., Guichard E., Salles C. (2014). Salt and fat contents influence the microstructure of model cheeses, chewing/swallowing and in vivo aroma release. Flavour Fragr. J..

[112] Arancibia C., Jublot L., Costell E., Bayarri S. (2011). Flavor release and sensory characteristics of o/w emulsions. Influence of composition, microstructure and rheological behavior. Food Res. Int..

[113] Overington A.R., Eyres G.T., Delahunty C.M., Silcock P., Niimi J., Holland R., Coolbear T. (2011). Flavour release and perception in cheese bases. Aust. J. Dairy Technol..

[114] Guichard E., Repoux M., Qannari E.M., Laboure H., Feron G. (2017). Model cheese aroma perception is explained not only by in vivo aroma release but also by salivary composition and oral processing parameters. Food Funct..

[115] Hollowood T.A., Linforth R.S.T., Taylor A.J., Roberts D.D., Taylor A.J. (2000). The relationship between carvone release and the perception of mintyness in gelatin gels. Flavor Release.

[116] Overbosch P., Afterof W.G.M., Haring P.G.M. (1991). Flavor release in the mouth. Food Rev. Int..

[117] Linforth R.S.T., Baek I., Taylor A.J. (1999). Simultaneous instrumental and sensory analysis of volatile release from gelatine and pectin/gelatine gels. Food Chem..

[118] Brauss M.S., Linforth R.S.T., Cayeux I., Harvey B., Taylor A.J. (1999). Altering the fat content affects flavor release in a model yogurt system. J. Agric. Food Chem..

[119] Shojaei Z.A., Linforth R.S.T., Hort J., Hollowood T., Taylor A.J. (2006). Measurement and manipulation of aroma delivery allows control of perceived fruit flavour in low- and regular-fat milks. Int. J. Food Sci. Technol..

[120] Frank D., Appelqvist I., Piyasiri U., Wooster T.J., Delahunty C. (2011). Proton Transfer Reaction Mass Spectrometry and Time Intensity Perceptual Measurement of Flavor Release from Lipid Emulsions Using Trained Human Subjects. J. Agric. Food Chem..

[121] Miettinen S.-M., Hyvönen L., Linforth R.S.T., Taylor A.J., Tuorila H. (2004). Temporal aroma delivery from milk systems containing 0-5% added fat, observed by free choice profiling, time intensity, and atmospheric pressure chemical ionization-mass spectrometry techniques. J. Agric. Food Chem..

[122] Burseg K., Linforth R.S.T., Hort J., Taylor A.J. (2009). Flavor Perception in Biscuits; Correlating Sensory Properties with Composition, Aroma Release, and Texture. Chemosens. Percept..

[123] Baek I., Linforth R.S.T., Blake A., Taylor A.J. (1999). Sensory perception is related to the rate of change of volatile concentration in-nose during eating of model gels. Chem. Senses.

[124] Weel K.G.C., Boelrijk A.E.M., Alting A.C., vanMil P.J.J.M., Burger J.J., Gruppen H., Voragen A.G.J., Smit G. (2002). Flavor release and perception of flavored whey protein gels: Perception is determined by texture rather than by release. J. Agric. Food Chem..

[125] Déléris I., Saint-Eve A., Dakowski F., Sémon E., Le Quéré J.-L., Guillemin H., Souchon I. (2011). The dynamics of aroma release during consumption of candies of different structures, and relationship with temporal perception. Food Chem..

[126] Saint-Eve A., Martin N., Guillemin H., Sémon E., Guichard E., Souchon I. (2006). Flavored Yogurt Complex Viscosity Influences Real-Time Aroma Release in the Mouth and Sensory Properties. J. Agric. Food Chem..

[127] Lethuaut L., Weel K.G.C., Boelrijk A.E.M., Brossard C.D. (2004). Flavor perception and aroma release from model dairy desserts. J. Agric. Food Chem..

[128] Davidson J.M., Linforth R.S.T., Hollowood T.A., Taylor A.J. (1999). Effect of sucrose on the perceived flavor intensity of chewing gum. J. Agric. Food Chem..

[129] Saint-Eve A., Deleris I., Aubin E., Semon E., Feron G., Rabillier J.-M., Ibarra D., Guichard E., Souchon I. (2009). Influence of Composition (CO_2_ and Sugar) on Aroma Release and Perception of Mint-Flavored Carbonated Beverages. J. Agric. Food Chem..

[130] Salles C., Hollowood T.A., Linforth R.S.T., Taylor A.J., Le Quere J.L., Etiévant P.X. (2003). Relating real time flavour release to sensory perception of soft cheeses. Flavour Research at the Dawn of the Twenty-First Century.

[131] Déléris I., Saint-Eve A., Guo Y., Lieben P., Cypriani M.-L., Jacquet N., Brunerie P., Souchon I. (2011). Impact of Swallowing on the Dynamics of Aroma Release and Perception during the Consumption of Alcoholic Beverages. Chem. Senses.

[132] Charles M., Romano A., Yener S., Barnabà M., Navarini L., Märk T.D., Biasioli F., Gasperi F. (2015). Understanding flavour perception of espresso coffee by the combination of a dynamic sensory method and in-vivo nosespace analysis. Food Res. Int..

[133] Deuscher Z. (2019). Identifier Les Marqueurs Clés de la Qualité Organoleptique des Chocolats Pour Prédire Leurs Caractéristiques Sensorielles. Ph.D. Thesis.

[134] Deuscher Z., Andriot I., Sémon E., Cordelle S., Schlich P., Repoux M., Roger J.M., Boulanger R., Labouré H., Le Quéré J.L., Hansel A., Dunkl J. (2019). Dark chocolates organoleptic differences: A PTR-ToF-MS success story. Proceedings of the 8th International Conference on Proton Transfer Reaction Mass Spectrometry and its Applications.

[135] Deuscher Z., Andriot I., Cordelle S., Repoux M., Boulanger R., Labouré H., Schlich P., Le Quéré J.L. Nosespace of dark chocolates differing in sensory characteristics using PTR-TOF-MS and link to flavour perception through simultaneous Temporal Dominance of Sensations (TDS). Proceedings of the 12th Wartburg Symposium on Flavor Chemistry & Biology.

[136] Peltier C., Visalli M., Labouré H., Hélard C., Andriot I., Cordelle S., Le Quéré J.-L., Schlich P. (2022). Automatic pre-treatment and multiblock analysis of flavor release and sensory temporal data simultaneously collected in vivo. J. Chemometr..

[137] Pedrotti M., Spaccasassi A., Biasioli F., Fogliano V. (2019). Ethnicity, gender and physiological parameters: Their effect on in vivo flavour release and perception during chewing gum consumption. Food Res. Int..

[138] van Eck A., Pedrotti M., Brouwer R., Supapong A., Fogliano V., Scholten E., Biasioli F., Stieger M. (2021). In Vivo Aroma Release and Dynamic Sensory Perception of Composite Foods. J. Agric. Food Chem..

[139] Gonzalez-Estanol K., Khomenko I., Cliceri D., Biasioli F., Stieger M. (2023). In vivo aroma release and perception of composite foods using nose space PTR–ToF–MS analysis with Temporal-Check-All-That-Apply. Food Res. Int..

[140] Pittari E., Piombino P., Andriot I., Cheynier V., Cordelle S., Feron G., Gourrat K., Le Quéré J.-L., Meudec E., Moio L. (2022). Effects of oenological tannins on aroma release and perception of oxidized and non-oxidized red wine: A dynamic real-time in-vivo study coupling sensory evaluation and analytical chemistry. Food Chem..

[141] Yang N., Yang Q., Chen J., Fisk I. (2021). Impact of capsaicin on aroma release and perception from flavoured solutions. LWT-Food Sci. Technol..

[142] Chen X., Zhang W., Quek S.Y., Zhao L. (2023). Flavor–food ingredient interactions in fortified or reformulated novel food: Binding behaviors, manipulation strategies, sensory impacts, and future trends in delicious and healthy food design. Comp. Rev. Food Sci. Food Saf..

[143] de la Fuente Blanco A., Sáenz-Navajas M.-P., Ballester J., Franco-Luesma E., Valentin D., Ferreira V. (2023). Sensory dimensions derived from competitive and creative perceptual interactions between fruity ethyl esters and woody odorants in wine-like models. OENO One.

[144] Ma Y., Guibert A., Béno N., Tang K., Xu Y., Thomas-Danguin T. (2023). Exploring the effects of mixture composition factors and perceptual interactions on the perception of icewine odor: An olfactometer-based study. Food Chem..

[145] Yuan J., Anantharamkrishnan V., Hoye T.R., Reineccius G.A. (2023). Covalent Adduct Formation between β-Lactoglobulin and Flavor Compounds under Thermal Treatments That Mimic Food Pasteurization or Sterilization. J. Agric. Food Chem..

[146] Gierczynski I., Guichard E., Laboure H. (2011). Aroma perception in dairy products: The roles of texture, aroma release and consumer physiology. A review. Flavour Fragr. J..

[147] Bult J.H.F., de Wijk R.A., Hummel T. (2007). Investigations on multimodal sensory integration: Texture, taste, and ortho- and retronasal olfactory stimuli in concert. Neurosci. Lett..

[148] Relkin P., Fabre M., Guichard E. (2004). Effect of fat nature and aroma compound hydrophobicity on flavor release from complex food emulsions. J. Agric. Food Chem..

[149] Boisard L., Andriot I., Martin C., Septier C., Boissard V., Salles C., Guichard E. (2014). The salt and lipid composition of model cheeses modifies in-mouth flavour release and perception related to the free sodium ion content. Food Chem..

[150] Salles C., Voilley A., Etiévant P. (2006). Odour-taste interactions in flavour perception. Flavour in Food.

[151] Voilley A., Etiévant P. (2006). Flavour in Food.

[152] Guichard E., Salles C., Morzel M., Le Bon A.-M. (2017). Flavour: From Food to Perception.

[153] Guichard E., Salles C. (2022). Flavor: From Food to Behaviors, Wellbeing and Health.

[154] Goubet I., Le Quéré J.L., Voilley A.J. (1998). Retention of aroma compounds by carbohydrates: Influence of their physicochemical characteristics and of their physical state. A review. J. Agric. Food Chem..

[155] Noble A.C. (1996). Taste-aroma interactions. Trends Food Sci. Technol..

[156] Green B.G., Nachtigal D., Hammond S., Lim J. (2012). Enhancement of retronasal odors by taste. Chem. Senses.

[157] Nasri N., Beno N., Septier C., Salles C., Thomas-Danguin T. (2011). Cross-modal interactions between taste and smell: Odour-induced saltiness enhancement depends on salt level. Food Qual. Pref..

[158] Spence C. (2022). Factors affecting odour-induced taste enhancement. Food Qual. Pref..

[159] Thomas-Danguin T., Guichard E., Salles C. (2019). Cross-modal interactions as a strategy to enhance salty taste and to maintain liking of low-salt food: A review. Food Funct..

[160] Lyu J., Chen S., Nie Y., Xu Y., Tang K. (2021). Aroma release during wine consumption: Factors and analytical approaches. Food Chem..

[161] Trimmer C., Keller A., Murphy N.R., Snyder L.L., Willer J.R., Nagai M.H., Katsanis N., Vosshall L.B., Matsunami H., Mainland J.D. (2019). Genetic variation across the human olfactory receptor repertoire alters odor perception. Proc. Natl. Acad. Sci. USA.

[162] Feron G., Ayed C., Qannari E.M., Courcoux P., Laboure H., Guichard E. (2014). Understanding aroma release from model cheeses by a statistical multiblock approach on oral processing. PLoS ONE.

[163] Schwartz M., Canon F., Feron G., Neiers F., Gamero A. (2021). Impact of Oral Microbiota on Flavor Perception: From Food Processing to In-Mouth Metabolization. Foods.

[164] Muñoz-González C., Brule M., Martin C., Feron G., Canon F. (2022). Molecular mechanisms of aroma persistence: From noncovalent interactions between aroma compounds and the oral mucosa to metabolization of aroma compounds by saliva and oral cells. Food Chem..

[165] Boichot V., Muradova M., Nivet C., Proskura A., Heydel J.-M., Canivenc-Lavier M.-C., Canon F., Neiers F., Schwartz M. (2022). The role of perireceptor events in flavor perception. Front. Food Sci. Technol..

[166] Buettner A., Beer A., Hannig C., Settles M. (2001). Observation of the swallowing process by application of videofluoroscopy and real-time magnetic resonance imaging—Consequences for retronasal aroma stimulation. Chem. Senses.

[167] Linforth R., Taylor A.J. (2000). Persistence of volatile compounds in the breath after their consumption in aqueous solutions. J. Agric. Food Chem..

[168] Labouré H., Repoux M., Courcoux P., Feron G., Guichard E. (2014). Inter-individual retronasal aroma release variability during cheese consumption: Role of food oral processing. Food Res. Int..

[169] Ruijschop R.M.A.J., Burgering M.J.M., Jacobs M.A., Boelrijk A.E.M. (2009). Retro-Nasal Aroma Release Depends on Both Subject and Product Differences: A Link to Food Intake Regulation?. Chem. Senses.

[170] Muñoz-González C., Martín-Álvarez P.J., Moreno-Arribas M.V., Pozo-Bayón M.Á. (2014). Impact of the Nonvolatile Wine Matrix Composition on the In Vivo Aroma Release from Wines. J. Agric. Food Chem..

[171] Ijichi C., Wakabayashi H., Sugiyama S., Ihara Y., Nogi Y., Nagashima A., Ihara S., Niimura Y., Shimizu Y., Kondo K. (2019). Metabolism of Odorant Molecules in Human Nasal/Oral Cavity Affects the Odorant Perception. Chem. Senses.

[172] Robert-Hazotte A., Faure P., Ménétrier F., Folia M., Schwartz M., Le Quéré J.-L., Neiers F., Thomas-Danguin T., Heydel J.-M. (2022). Nasal Odorant Competitive Metabolism Is Involved in the Human Olfactory Process. J. Agric. Food Chem..

[173] Small D.M., Prescott J. (2005). Odor/taste integration and the perception of flavor. Exp. Brain Res..

[174] Pu D., Zhang H., Zhang Y., Sun B., Ren F., Chen H., He J. (2019). Characterization of the aroma release and perception of white bread during oral processing by gas chromatography-ion mobility spectrometry and temporal dominance of sensations analysis. Food Res. Int..

[175] van Eck A., Stieger M. (2020). Oral processing behavior, sensory perception and intake of composite foods. Trends Food Sci. Technol..

